# Combining glucose and high-sensitivity cardiac troponin in the early diagnosis of acute myocardial infarction

**DOI:** 10.1038/s41598-023-37093-1

**Published:** 2023-09-05

**Authors:** Ana Yufera-Sanchez, Pedro Lopez-Ayala, Thomas Nestelberger, Karin Wildi, Jasper Boeddinghaus, Luca Koechlin, Maria Rubini Gimenez, Hüseyin Sakiz, Paolo Bima, Oscar Miro, F. Javier Martín-Sánchez, Michael Christ, Dagmar I. Keller, Danielle M. Gualandro, Damian Kawecki, Katharina Rentsch, Andreas Buser, Christian Mueller, The APACE Investigators

**Affiliations:** 1 Department of Cardiology, University Heart Center Basel, and Cardiovascular Research Institute Basel (CRIB), University Hospital Basel, University of Basel, Basel, Switzerland; 2 GREAT Network, Basel, Switzerland; 3 Department of Intensive Care, University Hospital Basel, University of Basel, Basel, Switzerland; 4 Department of Cardiology, Royal Infirmary of Edinburgh, Edinburgh, UK; 5 Cardiology Department, Heart Center Leipzig, Leipzig, Germany; 6 Department of Cardiac Surgery, University Hospital Basel, University of Basel, Basel, Switzerland; 7 Department of Medical Sciences, University of Turin, Turin, Italy; 8 Emergency Department, Hospital Clinic, Barcelona, Catalonia Spain; 9 Emergency Department, Hospital Clínico San Carlos, Madrid, Spain; 10 Department of Emergency Medicine, Luzerner Kantonsspital, Luzern, Switzerland; 11 Emergency Department, University Hospital Zurich, Zurich, Switzerland; 12 2nd Department of Cardiology, School of Medicine in Zabrze, Medical University of Sielsia, Katowice, Poland; 13 Laboratory Medicine, University Hospital Basel, University of Basel, Basel, Switzerland; 14 Blood Transfusion Centre, Swiss Red Cross, Basel, Switzerland; 15 Department of Hematology, University Hospital Basel, University of Basel, Basel, Switzerland

**Keywords:** Cardiology, Biomarkers, Diagnostic markers, Prognostic markers

## Abstract

Glucose is a universally available inexpensive biomarker, which is increased as part of the physiological stress response to acute myocardial infarction (AMI) and may therefore help in its early diagnosis. To test this hypothesis, glucose, high-sensitivity cardiac troponin (hs-cTn) T, and hs-cTnI were measured in consecutive patients presenting with acute chest discomfort to the emergency department (ED) and enrolled in a large international diagnostic study (NCT00470587). Two independent cardiologists centrally adjudicated the final diagnosis using all clinical data, including serial hs-cTnT measurements, cardiac imaging and clinical follow-up. The primary diagnostic endpoint was index non-ST-segment elevation MI (NSTEMI). Prognostic endpoints were all-cause death, and cardiovascular (CV) death or future AMI, all within 730-days. Among 5639 eligible patients, NSTEMI was the adjudicated final diagnosis in 1051 (18.6%) patients. Diagnostic accuracy quantified using the area under the receiver-operating characteristics curve (AUC) for the combination of glucose with hs-cTnT and glucose with hs-cTnI was very high, but not higher versus that of hs-cTn alone (glucose/hs-cTnT 0.930 [95% CI 0.922–0.937] versus hs-cTnT 0.929 [95% CI 0.922–0.937]; glucose/hs-cTnI 0.944 [95% CI 0.937–0.951] versus hs-cTnI 0.944 [95% CI 0.937–0.951]). In early-presenters, a dual-marker strategy (glucose < 7 mmol/L and hs-cTnT < 5/hs-cTnI < 4 ng/L) provided very high and comparable sensitivity to slightly lower hs-cTn concentrations (cTnT/I < 4/3 ng/L) alone, and possibly even higher efficacy. Glucose was an independent predictor of 730-days endpoints. Our results showed that a dual marker strategy of glucose and hs-cTn did not increase the diagnostic accuracy when used continuously. However, a cutoff approach combining glucose and hs-cTn may provide diagnostic utility for patients presenting ≤ 3 h after onset of symptoms, also providing important prognostic information.

## Introduction

Despite significant improvements in its diagnosis and treatment, acute myocardial infarction (AMI) remains one of the leading causes of death worldwide^1–3^. Early diagnosis of AMI is crucial for initiating evidence-based therapy, which has been shown to be highly effective when promptly initiated^4^. As most patients presenting to the emergency department (ED) with acute chest discomfort will ultimately be diagnosed with other and often rather benign causes of chest discomfort, also the rapid and safe identification of these low-risk patients is of great medical and economic importance. Early detection of these patients can help reduce the length of ED stay and avoid unnecessary hospitalization and testing^2,5–7^.

The development of high sensitivity cardiac troponin (hs-cTn) assays and rapid hs-cTn-based triage algorithms significantly increased the diagnostic accuracy for AMI and reduced the time to diagnosis^2,5–7^. Currently, the most extensively validated strategy is the ESC hs-cTn-0/1 h-algorithm (class I recommendation)^2^, which combines the hs-cTn concentration at presentation with 0/1 h absolute changes in hs-cTn to triage patients towards rule-out or rule-in^2^. Increasing the percentage of patients in whom safe rule-out or safe rule-in could be achieved already using the information obtained from the 0 h (ED presentation) blood draw only and thereby avoiding the logistic challenges and extra time need for the 1 h blood draw would be very desirable both from a medical and an economic perspective.

Pilot data had suggested that glucose, as a low-cost, widely available biomarker, which is commonly increased in patients with diabetes mellitus, who have a much higher risk of AMI, but also during acute physiological stress situations such as AMI^8^, could further increase the diagnostic accuracy of the hs-cTn concentration at ED presentation^9–12^. We therefore aimed to evaluate in a large prospective diagnostic study using central adjudication whether glucose provides incremental (1) diagnostic value to hs-cTnT or hs-cTnI for diagnosing AMI, or (2) prognostic value of death or future AMI.

## Methods

### Study design and patient population

This was a secondary analysis from a large prospective multicentre international diagnostic study (NCT00470587)^7,13–15^ recruiting adult patients presenting to the ED with acute chest discomfort/pain. While recruitment was independent from renal function at presentation, patients with end-stage renal failure on chronic dialysis were excluded. Patients were also excluded for this analysis if (A) they presented with ST-segment elevation myocardial infarction (STEMI), (B) the final diagnosis remained unclear even after final adjudication and had at least one elevated hs-cTnT concentration, possibly indicating the presence of AMI, (C) they presented with chest pain onset at maximum > 12 h, and (D) hs-cTnT, hs-cTnI or routine non-fasting plasma glucose concentrations at ED presentation were not available (Supplementary Fig. S1).

The study was carried out according to the principles of the Declaration of Helsinki and was approved by the ethics committee EKBB (Ethikkomission beider Basel; Ref. Nr. EK 280/05) and local ethics committees. Written informed consent was obtained from all participating patients. The authors designed the study, gathered, and analysed the data according to the STROBE (Strengthening the Reporting of Observational Studies in Epidemiology) guidelines^16^ (Supplementary Table S1). Additional information regarding blood samplings, laboratory methods and follow-up can be found in the Supplementary Material.

### Adjudicated final diagnosis

Adjudication of the final diagnosis was performed centrally at the core laboratory by two independent cardiologists according to current guidelines and the fourth universal definition of MI^1,2^. The details of the adjudication have been previously reported^7,13–15^.

All hs-cTnT-based analyses were carried out using an adjudication including serial hs-cTnT blood concentrations. Accordingly, all hs-cTnI-based analyses were carried out using an adjudication including serial hs-cTnI blood concentrations. Doing so, a possible underestimation of the actual performance of hs-cTnI-based algorithms due to possible mismatching results for hs-cTnT and hs-cTnI was avoided. Although rare, this phenomenon has been previously observed^17^.

### Clinical endpoints

The primary diagnostic endpoint was non-ST-segment elevation myocardial infarction (NSTEMI) during the index hospitalization. The co-primary prognostic endpoint was all-cause death as well as the composite of cardiovascular death or AMI excluding the index event within 30 days and 2 years from ED presentation.

### Glucose cut-offs and accelerated diagnostic pathways

It is currently unknown, which glucose concentrations should be used for the help for rule-out or rule-in of NSTEMI. Therefore, several previously used thresholds were tested. The American Diabetes Association (ADA)^18^ cut-off for impaired fasting glucose (≥ 5.6 mmol/L), was selected for the main analysis, as this value was most frequently used previously in this indication^9,10,19^. In addition, two further rule-out cut-offs were assessed: 6.1 mmol/L (WHO threshold for impaired fasting glucose)^20^ and 7 mmol/L (threshold for pathologic plasma glucose concentrations if measured in fasting, according to both the WHO and the ADA)^18,20^. For rule-in, we explored different cut-offs that were defined as abnormal glucose values in current diabetes guidelines^18,20^ as well as thresholds that had previously been used to either define stress hyperglycaemia or for rule-in of NSTEMI: glucose ≥ 7 mmol/L (ADA’s threshold for diabetes diagnosis if measured in fasting), ≥ 7.8 mmol/L (ADA and WHO’s pathologic threshold for the oral glucose tolerance test) or ≥ 11.1 mmol/L (ADA’s threshold for diabetes diagnosis in a random glucose sample)^21–23^.

The aforementioned cut-offs were assessed individually as well as in combination with the ESC hs-cTn single cut-off rule-out (hs-cTnT < 5 ng/L and hs-cTnI < 4 ng/L) and rule-in (hs-cTnT ≥ 52 ng/L and hs-cTnI ≥ 64 ng/L) strategies in patients who presented more than 3 h after onset of symptoms, resulting in a dual-biomarker strategy (admission hs-cTn and glucose concentrations). The dual-biomarker strategy was further tested in early presenters (defined as patients presenting to the ED ≤ 3 h after onset of symptoms), a population in whom current ESC guidelines do not recommend the use of a hs-cTn-only approach using 0 h-samples for rule-out^2,5,6^. As the release of hs-cTn as a structural protein from injured cardiomyocytes in AMI is a time-dependent phenomenon, even with the use of hs-cTn assays some time-delay from the onset of cardiomyocyte injury to a relevant increase in the systemic circulation seems to remain^2,5,6^. Lower hs-cTn thresholds (hs-cTnT < 4 ng/L, hs-cTnI < 3 ng/L) were also assessed, as they might provide higher safety and reduce the number of false negatives in this subgroup of patients for whom there is currently no validated approach for an early rule-out for NSTEMI. In addition, the optimal rule-out and rule-in glucose cut-offs were tested in combination with the European Society of Cardiology (ESC) 0/1 h-hs-cTn algorithm (Supplementary Fig. S2) and its diagnostic performance was directly compared to that provided by the ESC 0/1 h algorithm alone.

### Statistical analysis

Continuous variables are reported as medians and interquartile range (IQR) and compared with the use of the Mann–Whitney U test. Categorical variables are reported as numbers and percentages and compared with the use of the Pearson chi-square test or Fisher exact test, as appropriate. Confidence intervals (CIs) of proportions were computed according to Wilson’s method^24^.

Diagnostic accuracy of continuous concentrations of hs-cTn alone and combined with glucose, respectively, was quantified by using the Area Under the Receiver-Operating-Characteristic curves (AUC). Comparison of AUC’s 95% confidence intervals (95% CI) and their p-values was performed as recommended by DeLong^25^. As hs-cTn single cut-offs strategies for ruling out AMI are currently recommended for clinical use only in patients presenting > 3 h after chest pain onset (CPO)^2,5,6^, subgroup analyses were conducted for early presenters (CPO ≤ 3 h) to investigate whether the combination of hs-cTn with glucose might allow safe rule-out of NSTEMI also in this challenging patient group. Additional subgroup analyses were performed in patients with intermediate hs-cTn concentrations (defined as hs-cTn concentrations within the observe zone of the ESC 0/1 h-algorithm), and patients with and without diabetes. In a sensitivity analysis the diagnostic added value of glucose was individually assessed for type1 and type 2 NSTEMI. To study the diagnostic performance of the different glucose cut-offs and their combination with the accelerated diagnostic pathways, safety was assessed as the sensitivity and negative predictive value (NPV) of ruling-out index NSTEMI, accuracy as the specificity and positive predictive value (PPV) of ruling-in index NSTEMI, and efficacy was quantified by the proportion of patients triaged toward rule-out or rule-in of NSTEMI upon admission to the ED. Sensitivity and specificity were compared by means of the McNemar test for paired proportions, while NPV and PPV were tested using a weighted generalised score statistic^26^.

Survival as well as the composite of cardiovascular death and AMI for 30-days and 730-days follow-up were plotted in Kaplan–Meier curves and the log rank test was used to assess differences between groups (Supplementary Methods). In a multivariable adjusted analysis, a cox proportional hazards model was fitted to assess the possible association between glucose concentration and our prognostic endpoints. To avoid dichotomizing glucose into a few discrete, ordered levels and to avoid imposing linearity we used a restricted cubic spline function to model the continuous non-linear association between glucose and the outcome^27^. Three knots were placed at the 0.1, 0.5 and 0.9 percentiles of the marginal distribution of the biomarker, as recommended by Harrell^28^. Considering the total number of events for each outcome and to avoid overfitting in the model, two multivariable models were built for prognostic purposes. The variables in the models were selected based on clinical knowledge and previous literature, regardless of the p-value. Model A included age, malignancy, diabetes mellitus and eGFR. Model B included age, sex, diabetes mellitus, eGFR, malignancy, BMI, hypercholesterolemia, hypertension, and active smoking status. The proportional hazard assumption was confirmed using the Schoenfield residuals test. The magnitude of the effect of glucose was graphically assessed (dose–response plots). To compute the hazard ratio, a reference value of 5.6 mmol/L was chosen, in accordance with the cut-off used for triaging towards rule-out. To assess whether the presence of diabetes could be an effect modifier of glucose, in a secondary analysis we fitted an interaction between glucose and diabetes (Supplementary Methods). All hypothesis testing was two-tailed, and p-values below 0.05 were considered statistically significant. Statistical analyses were performed in SPSS for Windows 25.0 (SPSS Inc., Chicago, IL) and R, Version 3.6.3 (The R Foundation).

## Results

### Patient characteristics

Among 5639 eligible patients with available hs-cTnT, hs-cTnI, and random glucose concentrations at ED presentation (Supplementary Fig. S1), NSTEMI was the adjudicated final diagnosis in 1051 (18.6%) patients. The median age was 61 years (interquartile range [IQR] 49 to 74) and 33.5% of patients were women (Table 1).

**Table 1:** Baseline characteristics according to their glucose concentrations.

	Total cohort	Glucose < 5.6 mmol/L	Glucose $\ge$ 5.6 mmol/L	p-values
	n = 5639	n = 1785 (31.7%)	n = 3854 (68.3%)	
Descriptive factors				
Women-no (%)	1889 (33.5)	660 (37.0)	1229 (31.9)	0.001
Age, median (IQR)	61 (49–74)	54 (42–69)	63 (52–75)	< 0.001
BMI, median (IQR)	26.5 (23.9–29.7)	25.7 (23.1–28.7)	26.9 (24.2–30.1)	< 0.001
Chest pain onset, median (IQR)	5 (2–12)	5 (2–14.5)	5 (2–12)	0.124
Early presenters-no (%)	2093 (37.1%)	650 (36.4%)	1443 (37.4%)	0.460
Vital parameters, median IQR				
Systolic BP-mmHg	140 (125–156)	137 (123–153)	141 (127–157)	< 0.001
Diastolic BP-mmHg	80 (71–90)	81 (72–90)	80 (71–90)	0.234
Heart rate-bpm	76 (66–89)	73 (65–85)	77 (67–90)	< 0.001
Risk factors-no (%)				
Hypertension	3378 (59.9)	818 (45.8)	2560 (66.4)	< 0.001
Hypercholesterolemia	2672 (47.4)	667 (37.4)	2005 (52.0)	< 0.001
Diabetes mellitus	982 (17.3)	89 (5.0)	893 (23.2)	< 0.001
Active smoker	1397 (24.8)	498 (27.9)	899 (23.3)	< 0.001
Patients history-no (%)				
Coronary artery disease	1834 (32.5)	427 (23.9)	1407 (36.5)	< 0.001
Prior AMI	1310 (23.2)	295 (16.5)	1015 (26.3)	< 0.001
Prior revascularization	1538 (27.3)	372 (20.8)	1166 (30.3)	< 0.001
Prior stroke	297 (5.3)	75 (4.2)	222 (5.8)	0.015
Peripheral artery disease	298 (5.3)	56 (3.1)	242 (6.3)	< 0.001
Medication at presentation-no (%)				
Platelet inhibitor	2155 (38.2)	507(28.4)	1648 (42.8)	< 0.001
Beta-blocker	1903 (33.7)	435 (24.4)	1468 (38.1)	< 0.001
ACE-inhibitor and ARB	2220 (39.4)	499 (28.0)	1721 (44.7)	< 0.001
Statin	1977 (35.1)	444 (24.9)	1533 (39.8)	< 0.001
Calcium antagonists	852 (15.1)	177 (9.9)	675 (17.5)	< 0.001
Nitrates	543 (9.6)	124 (6.9)	419 (10.9)	< 0.001
Oral antidiabetics	538 (9.5)	46 (2.6)	492 (12.8)	< 0.001
Insulin	310 (5.5)	23 (1.3)	287 (7.4)	< 0.001
Blood test at presentation, median (IQR)				
Glucose-mmol/L	6.0 (5.4–7.2)	5.1 (4.8–5.4)	6.6 (6.0–8.0)	< 0.001
Hemoglobin-g/L	143 (132–153)	144 (133–154)	142 (131–153)	< 0.001
GFR MDRD-mL/min/1.73 m^2^	84.0 (68.4–100.0)	88.6 (73.7–103.6)	81.4 (65.9–98.1)	< 0.001
Hs-cTnT-ng/L	8.0 (4.0–19.0)	6.0 (3.0–12.0)	9.0 (5.0–24.0)	< 0.001
Hs-cTnI-ng/L	4.1 (2.0–15.0)	3.0 (2.0–8.0)	5.0 (2.0–20.0)	< 0.001

Mann–Whitney U test for continuous variables (not normal distributed), expressed in medians and interquartile range (IQR) and Chi-square test for categorical variables, expressed in numbers and percentages.

*Hs-cTnI* high sensitivity cardiac troponin I, *Hs-cTnT* high sensitivity cardiac troponin T, *SBP* systolic blood pressure, *DBP* diastolic blood pressure, *CAD* coronary artery disease, *AMI* acute myocardial infarction, *ACE-inhibitor* angiotensin-converting-enzyme inhibitor, *ARB* angiotensin receptor blocker, *GFR-MDRD* glomerular filtration rate-modification of diet in renal disease equation, *early presenters* patients with onset of symptoms of ≤ 3 h.

More than two-thirds of patients (68.3%) had random glucose concentrations ≥ 5.6 mmol/L at ED presentation. These patients were older, more often male, and more frequently had other cardiovascular risk factors, particularly diabetes mellitus (23% versus 5%) versus patients with glucose < 5.6 mmol/L. They also more often had established coronary artery disease (CAD) and history of a previous AMI or revascularization (p < 0.001). Accordingly, cardiovascular medication also differed between patients with higher glucose concentration and patients with concentrations below 5.6 mmol/L, with a much higher use of platelet inhibitors, beta-blockers, ACE-inhibitors or ARB or statins (all p < 0.001) in the former. These differences in baseline characteristics may have contributed to the higher hs-cTn concentration (9 vs 6 ng/L for hs-cTnT and 5 vs 3 ng/L for hs-cTnI) observed in the high glucose group (i.e. ≥ 5.6 mmol/L). There were no significant differences between groups regarding time to presentation after onset of symptoms.

### Final adjudicated diagnosis

**Figure 1:**
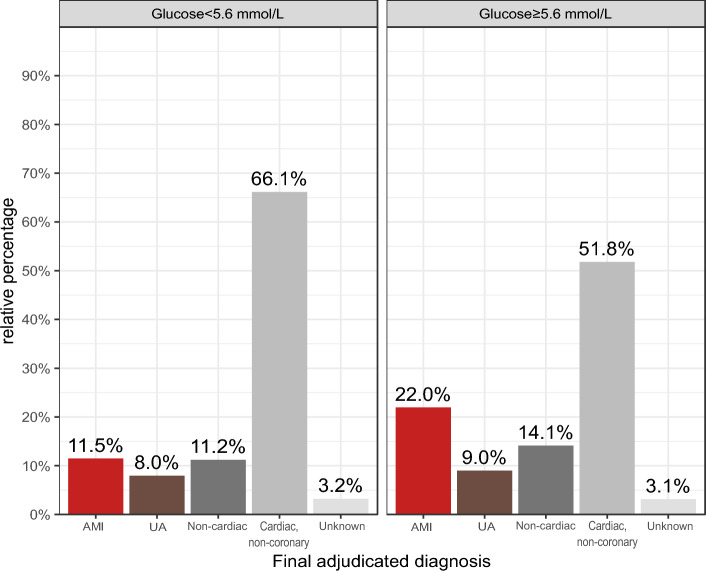
Bar diagram showing the rates of the different final adjudicated diagnosis according to their glucose concentration. *AMI* acute myocardial infarction, *UA* unstable angina.

1051 out of 5639 (18.6%) patients had an adjudicated final diagnosis of NSTEMI (Supplementary Table 2). Among 3854 patients with random glucose concentrations ≥ 5.6 mmol/L, the adjudicated final diagnosis was NSTEMI in 846 (22.0%); unstable angina in 346 (9.0%); cardiac symptoms of origin other than CAD such as takotsubo syndrome, tachyarrhythmia, heart failure, or myocarditis in 544 (14.1%); non-cardiac symptoms in 1997 (51.8%); and unknown diagnosis in 121 (3.1%) (Fig. 1).

### Glucose concentrations at presentation according to final diagnosis

Glucose concentrations were comparable (median 6.2 mmol/L) for patients with an adjudicated final diagnosis of unstable angina, cardiac, but non-coronary symptoms and patients with an unknown final diagnosis. Higher glucose concentrations were observed in patients with NSTEMI (median 6.6 mmol/L, IQR: 5.7 to 8.7 mmol/L), while patients that were found to have non-cardiac symptoms had a median glucose concentration of 5.8 mmol/L (IQR: 5.3 to 6.8 mmol/L) (Supplementary Fig. S3).

### Diagnostic performance of glucose

The diagnostic accuracy of glucose as a continuous variable for NSTEMI as quantified by the AUC was 0.635 (95% CI 0.617–0.654). The use of glucose < 5.6 mmol/L for early rule-out of NSTEMI in all-commers provided a sensitivity of 80.5% (95% CI 78.0–82.8) and a NPV of 88.5% (95% CI 87.0–89.9), missing 205 NSTEMI patients. Higher cutoffs (< 6.1 and < 7 mmol/L) substantially decreased the diagnostic performance (Supplementary Table 3). When applied only in non-early presenters, diagnostic performance did not improve significantly (Table 2). The use of glucose ≥ 11.1 mmol/L for rule-in of NSTEMI in all-commers, showed a specificity of 95.8% (95% CI 95.2–96.4%) and a PPV of 40.7% (95% CI 35.5–46.1%). Also here, using lower cut-offs substantially decreased diagnostic accuracy (Table 3).

**Table 2:** Direct comparison of the ESC hs-cTn 0 h cut-offs alone and their combination with different glucose cut-offs for rule-out of NSTEMI in patients presenting more than 3 h after chest pain onset (CPO).

Sensitivity/NPV analysis for the diagnosis of MI						
	Sensitivity	p-value	NPV	p-value	Patients ruled out	False negatives
Glucose < 5.6 mmol/L	80.4 (77.2–83.2)		88.5 (86.5–90.2)		1135	131
Glucose < 6.1 mmol/L	62.1 (58.4–65.7)		85.9 (84.2–87.4)		1788	253
Glucose < 7 mmol/L	40.9 (37.2–44.6)		84.5 (83.1–85.9)		2554	395
**Hs-cTnT < 5 ng/L (REFERENCE)**	100 (99.4–100.00)		100 (99.6–100.0)		913	0
Hs-cTnT < 5 ng/L + Glucose < 5.6 mmol/L	100 (99.4–100.00)	1.0	100 (99.1–100.00)	1.0	429	0
Hs-cTnT < 5 ng/L + Glucose < 6.1 mmol/L	100 (99.4–100.00)	1.0	100 (99.4–100.0)	1.0	591	0
Hs-cTnT < 5 ng/L + Glucose < 7 mmol/L	100 (99.4–100.00)	1.0	100 (99.5–100.0)	1.0	769	0
**Hs-cTnI < 4 ng/L (REFERENCE)**	99.8 (99.0–100.0)		99.9 (99.6–100.0)		1418	1
Hs-cTnI < 4 ng/L + Glucose < 5.6 mmol/L	100.0 (99.3–100.0)	0.317	100 (99.3–100.0)	0.524	574	0
Hs-cTnI < 4 ng/L + Glucose < 6.1 mmol/L	100.0 (99.3–100.0)	0.317	100 (99.5–100.0)	0.442	838	0
Hs-cTnI < 4 ng/L + Glucose < 7 mmol/L	100.0 (99.3–100.0)	0.317	100 (99.7–100.0)	0.372	1130	0

For hs-cTnT all patients with CPO > 3 h and with an adjudicated final diagnosis including serial hs-cTnT concentrations were taken into account (n = 3546); for hs-cTnI, all patients presenting more than 3 h after CPO and with an adjudicated final diagnosis including serial hs-cTnI concentrations (n = 3139).

p-values refer to the comparison of sensitivities and NPVs between the reference and the different dual-biomarker strategies.

**Table 3:** Direct comparison of the ESC hs-cTn 0 h cut-offs alone and their combination with different glucose cut-offs for rule-in of NSTEMI in all-commers.

Specificity/PPV analysis for the diagnosis of MI						
	Specificity	p-value	PPV	p-value	Patients ruled in	False positives
Glucose ≥ 7 mmol/L	75.2 (73.9–76.4)		28.4 (26.3–30.7)		1589	1137
Glucose ≥ 7.8 mmol/L	84.3 (83.2–85.3)		31.2 (28.5–34.1)		1047	720
Glucose ≥ 11.1 mmol/L	95.8 (95.2–96.4		40.7 (35.5–46.1)		322	191
**Hs-cTnT ≥ 52 ng/L (REFERENCE)**	97.2 (96.7–97.7)		80.0 (76.7–82.9)		634	127
Hs-cTnT ≥ 52 ng/L OR Glucose ≥ 7 mmol/L	73.6 (72.3–74.8)	< 0.001	37.9 (35.8–40.1)	< 0.001	1953	1213
Hs-cTnT ≥ 52 ng/L OR Glucose ≥ 7.8 mmol/L	82.3 (81.1–83.4)	< 0.001	45.5 (43.0–48.0)	< 0.001	1491	813
Hs-cTnT ≥ 52 ng/L OR Glucose ≥ 11.1 mmol/L	93.3 (92.5–94.0)	< 0.001	65.4 (62.2–68.4)	< 0.001	887	307
**Hs-cTnI ≥ 64 ng/L (REFERENCE)**	96.6 (96.0–97.1)		78.1 (74.7–81.2)		631	138
Hs-cTnI ≥ 64 ng/L OR Glucose ≥ 7 mmol/L	72.5 (71.1–73.9)	< 0.001	37.1 (34.9–39.4)	< 0.001	1796	1129
Hs-cTnI ≥ 64 ng/L OR Glucose ≥ 7.8 mmol/L	81.1 (79.9–82.3)	< 0.001	44.5 (41.9–47.1)	< 0.001	1397	775
Hs-cTnI ≥ 64 ng/L OR Glucose ≥ 11.1 mmol/L	92.4 (91.6–93.2)	< 0.001	63.8 (60.5–66.9)	< 0.001	861	312

For hs-cTnT all patients with an adjudicated final diagnosis including serial hs-cTnT concentrations (n = 5639) were taken into account; for hs-cTnI, all patients with an adjudicated final diagnosis including serial hs-cTnI concentrations (n = 4981).

P-values refer to the comparison between specificities and PPVs between the reference and the different dual-biomarker algorithms.

### Diagnostic accuracy of hs-cTnT, hs-cTnI and their combination with glucose for NSTEMI

The diagnostic accuracy of hs-cTnT and hs-cTnI concentrations as quantified by the AUC for the diagnosis of NSTEMI was very high: hs-cTnT 0.929 (95% CI 0.922 to 0.937) and hs-cTnI 0. 944 (95% CI 0.937 to 0.951) respectively. Adding glucose to hs-cTnT (0.930 [95% CI 0.922 to 0.937] p-value for comparison = 0.334) and hs-cTnI (0.944 [95% CI 0.937 to 0.951], p-value for comparison = 0.836) did not improve diagnostic accuracy (Supplementary Fig. S4A,B). Similar findings were obtained in all pre-defined subgroups (Supplementary Tables 4A,B), except for the subgroup of patients with intermediate hs-cTnI concentrations (≥ 4 ng/L and < 64 ng/L), in which the combination with glucose provided an AUC of 0.835 (95% CI 0.813–0.857), while hs-cTnI alone provided an AUC of 0.829 (95% CI 0.807–0.852), p = 0.035. Similar findings were observed when adding glucose to hs-cTnT (p = 0.057). When assessing NSTEMI Type 1 and Type 2 individually, glucose did not provide incremental value (Supplementary Table 5A,B).

### Adding glucose to hs-cTn single cut-off strategies

#### Rule-out in patients with CPO > 3 h

In the subgroup of patients presenting to the ED more than 3 h after CPO (n = 3546), the currently guideline-approved subgroup for single cut-off strategies, the ESC cut-off for hs-cTnT showed a sensitivity for NSTEMI of 100% (95% CI 99.4–100.0%) and a NPV of 100% with 913 (25.7%) patients ruled out and no false negatives. The combination of glucose < 5.6 mmol/L and hs-cTnT < 5 ng/L showed identical sensitivity and NPV values, however, the number of patients ruled out was substantially lower (n = 429, 12.1%) (Table 2). The combination of hs-cTnT with glucose concentrations < 6.1 and < 7 mmol/L also failed to improve the diagnostic performance (Table 2).

In the subgroup of patients presenting more than 3 h after CPO and with an adjudicated final diagnosis based on hs-cTnI concentrations (n = 3139), the ESC cut-off for hs-cTnI (< 4 ng/L) had a sensitivity of 99.8% (95% CI 99.0–100.0) and a NPV of 99.9% (95% CI 99.6–100.0%) with 1418 (45.2%) patients ruled out and one false negative. The combinations of glucose < 5.6, < 6.1 or < 7 mmol/L and hs-cTnI did not improve the diagnostic performance of hs-cTnI alone (for all three a sensitivity 100% [95% CI 99.3–100.0%], p = 0.317). The number of ruled-out patients decreased again substantially (Table 2).

#### Rule-out in patients with CPO ≤ 3 h

In the subgroup of patients with a time from CPO to ED presentation of 3 h or less (n = 2093), using hs-cTnT < 5 ng/L alone showed a sensitivity of 99.0% (95% CI 97.3–99.6%) and a NPV of 99.4% (95% CI 98.5–99.8%) with 665 (31.8%) of early presenters ruled out and 4 false negatives. The combination of glucose < 5.6 mmol/L and hs-cTnT < 5 ng/L resulted in a sensitivity of 99.5% (95% CI 98.1–99.9) and a NPV of 99.3% (95% CI 97.3–99.8) with 271 (12.9%) of early presenters ruled out and 2 false negatives. The combination of a higher glucose cutoff (< 7 mmol/L) and hs-cTnT < 5 ng/L seemed to best balance a very high safety (2 false negatives) with a reasonable efficacy of early presenters triaged to direct rule-out (n = 358 [17.1%]). Alternatively, lowering the hs-cTnT cut-off to < 4 ng/L resulted in a sensitivity of 99.7% (95% CI 98.5–100%; p = 0.083) and NPV of 99.8% (95% CI 98.7- 100%; p = 0.243), and ruled out 438 (20.9%) early presenters. The diagnostic performance of different combinations is summarized in Table 4.

**Table 4:** Direct comparison of the ESC hs-cTn 0 h cut-offs alone and their combination with different glucose cut-offs for rule-out of NSTEMI in early presenters (CPO ≤ 3 h).

Sensitivity/NPV analysis for the diagnosis of MI						
	Sensitivity	p-value	NPV	p-value	Patients ruled out	False negatives
Glucose < 5.6 mmol/L	80.7 (76.4–84.3)		88.6 (85.9–90.8)		650	74
Glucose < 6.1 mmol/L	66.3 (61.4–70.9)		87.7 (85.6–89.6)		1051	129
Glucose < 7 mmol/L	46.7 (41.8–51.7)		86.4 (84.5–88.0)		1496	204
**Hs-cTnT < 5 ng/L (REFERENCE)**	99.0 (97.3–99.6)		99.4 (98.5–99.8)		665	4
Hs-cTnT < 5 ng/L + Glucose < 5.6 mmol/L	99.5 (98.1–99.9)	0.157	99.3 (97.3–99.8)	0.681	271	2
Hs-cTnT < 5 ng/L + Glucose < 6.1 mmol/L	99.5 (98.1–99.9)	0.157	99.5 (98.3–99.9)	0.635	421	2
Hs-cTnT < 5 ng/L + Glucose < 7 mmol/L	99.5 (98.1–99.9)	0.157	99.6 (98.7–99.9)	0.312	547	2
Hs-cTnT < 4 ng/L	99.7 (98.5–100)	0.083	99.8 (98.7–100)	0.243	438	1
Hs-cTnT < 4 ng/L + Glucose < 5.6 mmol/L	100 (99.0–100)	0.045	100 (98.0–100)	0.282	191	0
Hs-cTnT < 4 ng/L + Glucose < 6.1 mmol/L	100 (99.0–100)	0.045	100 (98.6–100)	0.196	276	0
Hs-cTnT < 4 ng/L + Glucose < 7 mmol/L	100 (99.0–100)	0.045	100 (98.9–100)	0.141	358	0
**Hs-cTnI < 4 ng/L (REFERENCE)**	98.5 (96.4–99.3)		99.5 (98.7–99.8)		914	5
Hs-cTnI < 4 ng/L + Glucose < 5.6 mmol/L	99.7 (98.3–99.9)	0.045	99.7 (98.4–99.9)	0.475	342	1
Hs-cTnI < 4 ng/L + Glucose < 6.1 mmol/L	99.4 (97.8–99.8)	0.083	99.6 (98.7–99.9)	0.455	548	2
Hs-cTnI < 4 ng/L + Glucose < 7 mmol/L	99.4 (97.8–99.8)	0.083	99.7 (99.0–99.9)	0.206	720	2
Hs-cTnI < 3 ng/L	99.1 (97.3–99.7)	0.157	99.6 (98.8–99.9)	0.474	711	3
Hs-cTnI < 3 ng/L + Glucose < 5.6 mmol/L	100 (98.8–100)	0.025	100 (98.6–100)	0.227	265	0
Hs-cTnI < 3 ng/L + Glucose < 6.1 mmol/L	99.7 (98.3–99.9)	0.045	99.8 (98.7–100)	0.313	437	1
Hs-cTnI < 3 ng/L + Glucose < 7 mmol/L	99.7 (98.3–99.9)	0.045	99.8 (99.0–100)	0.175	574	1

For hs-cTnT all early presenters with an adjudicated final diagnosis including serial hs-cTnT concentrations were taken into account (n = 2093); for hs-cTnI, all early presenters with an adjudicated final diagnosis including serial hs-cTnI concentrations (n = 1842).

*NSTEMI* non-ST-elevation myocardial infarction, *hs-cTnT* high sensitivity cardiac troponin T, *hs-cTnI* high sensitivity cardiac troponin I, *NPV* negative predictive value, *PPV* positive predictive value.

In the subgroup of patients with a time from CPO to ED presentation of 3 h or less and with an adjudicated final diagnosis integrating hs-cTnI concentrations (n = 1842), a single hs-cTnI < 4 ng/L strategy showed a sensitivity of 98.5% (95% CI 96.4–99.3%) and a NPV of 99.5% (95% CI 98.7–99.8%) with 914 (49.6%) of early presenters ruled four and 5 false negatives.

The combination of glucose < 5.6 mmol/L and hs-cTnI < 4 ng/L resulted in a sensitivity of 99.7% (95% CI 98.3–99.9) and a NPV of 99.7% (95% CI 98.4–99.9) with 342 (18.6%) of early presenters ruled out and 1 false negative. The combination of a higher glucose cutoff (< 7 mmol/L) and hs-cTnI < 4 ng/L seemed to best balance a very high safety (2 false negatives) with a good efficacy of early presenters triaged to direct rule-out (n = 720 [39.1%]). Alternatively, lowering the hs-cTnI cut-off to < 3 ng/L resulted in a sensitivity of 99.1% (95% CI 97.3–99.7%; p = 0.157) and a NPV of 99.6% (95% CI: 98.8–99.9%; p = 0.474) with a reduction in the number of ruled out early presenters to 711 (38.6%).

#### Rule-in in all-commers

Rule-in algorithms with glucose significantly decreased both specificity and PPV (p < 0.001) when compared to ESC recommendations alone (hs-cTn and glucose ≥ 7, ≥ 7.8 or ≥ 11.1 mmol/L), and also decreased the number of ruled-in patients significantly (Table 3).

### Adding glucose to the ESC 0/1 h-algorithm

**Figure 2:**
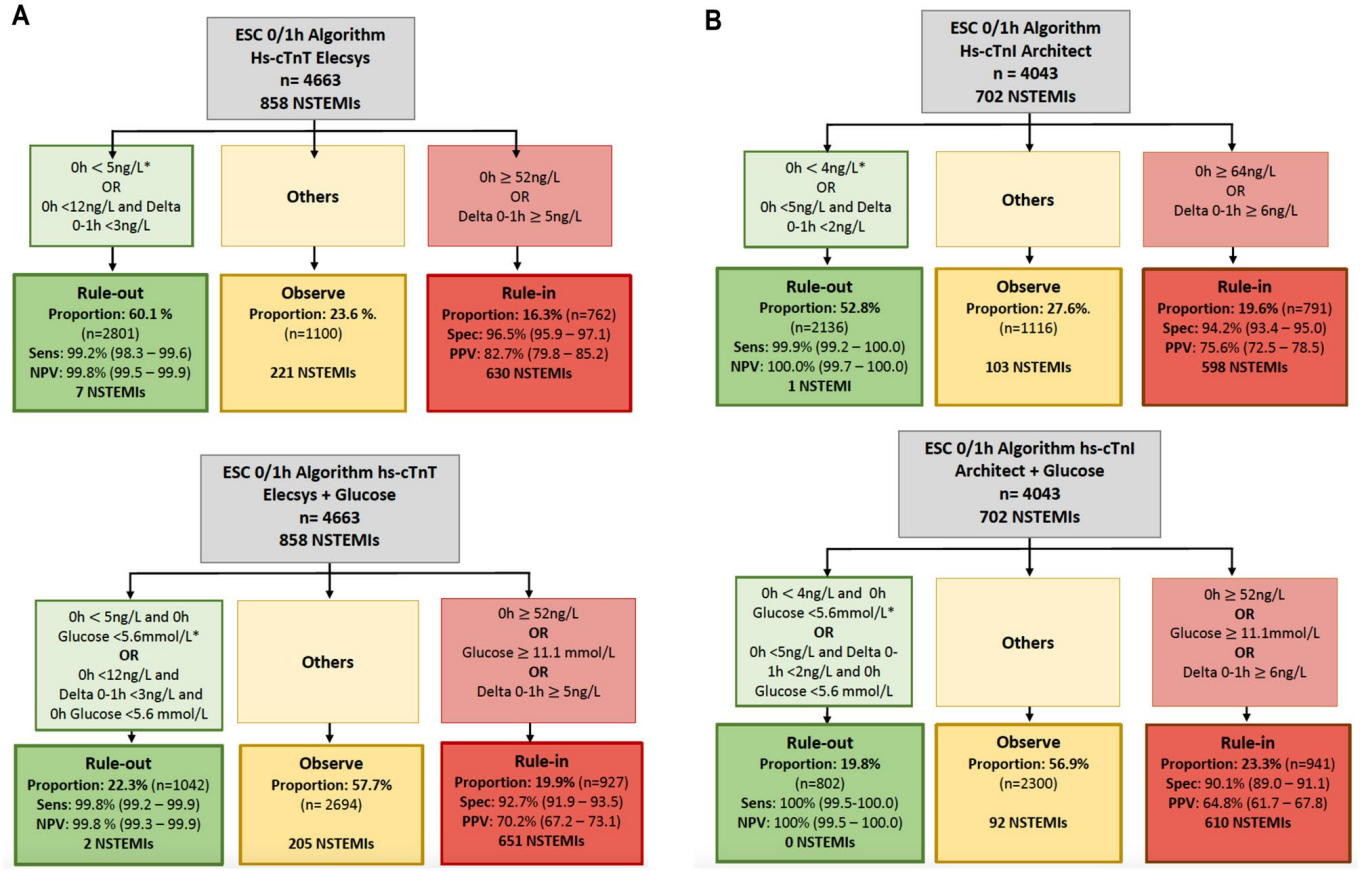
Direct comparison of the diagnostic performance of ESC hs-cTnT/hs-cTnI 0/1 h-algorithm and its combination with glucose concentration on admission for the early rule-out and rule-in of NSTEMI. (**A**) Shows a subgroup of 4663 patients with 0 and 1 h hs-cTnT concentrations available. (**B**) Shows a subgroup of 4043 patients with available 0 and 1 h hs-cTnI concentrations and in which the final diagnosis adjudication was done according to hs-cTnI values. *Only applicable if chest pain onset > 3 h. *NSTEMI* non-ST-Elevation myocardial infarction, *hs-cTnT* high sensitivity cardiac troponin T, *hs-cTnI* high sensitivity cardiac troponin I.

In the subgroup of 4663 patients with 0 and 1 h hs-cTnT concentrations available, the ESC hs-cTnT 0/1-algorithm triaged 60.1% patients towards rule-out of NSTEMI resulting in a sensitivity and NPV for index NSTEMI of 99.2% (95% CI 98.3–99.6%) and 99.8% (95% CI 99.5–99.9%), respectively. The ESC 0/1 h-algorithm triaged 16.3% patients towards rule-in with a specificity of 96.5% (95% CI 95.9–97.1%) and a PPV of 82.7% (95% CI 79.8–85.2%). Adding glucose < 5.6 mmol/L slightly increased sensitivity for rule-out to 99.8% (95% CI 99.2–99.9) with a difference in sensitivities of 0.6%, (p = 0.025), however it substantially reduced the number of ruled out patients (22.3% versus 60.1%, p < 0.001) (Fig. 2A).

In the subgroup of 4043 patients with available 0 and 1 h hs-cTnI concentrations and in which the final diagnosis adjudication was done according to hs-cTnI values, the ESC hs-cTnI 0/1-algorithm triaged 52.8% patients towards rule-out of NSTEMI resulting in a sensitivity and NPV of 99.9% (95% CI 99.2–100%) and 100% (95% CI 99.7–100%), respectively. A total of 19.6% patients were triaged towards rule-in with a specificity of 94.2% (95% CI 93.4–95.0%) and a PPV of 75.6% (95% CI 72.5–78.5%). Adding glucose < 5.6 mmol/L didn’t improve rule-out sensitivity (100% CI 99.5–100%) with a difference in sensitivities of 0.1 (p = 0.317) but reduced the number of ruled out patients (19.8% versus 52.8%, p < 0.001) (Fig. 2B).

Adding glucose $\ge$ 11.1 mmol/L significantly reduced specificity and PPV for rule-in for both hs-cTnT (92.7% [95% CI 91.9–93.5%], p < 0.001 and 70.2% [95% CI 67.2–73.1%], p < 0.001, respectively), and hs-cTnI (90.1% [95% CI 89.0–91.1%], p < 0.001 and 64.8% [95% CI 61.7–67.8%], p < 0.001, respectively).

### Prognostic performance of glucose

**Figure 3:**
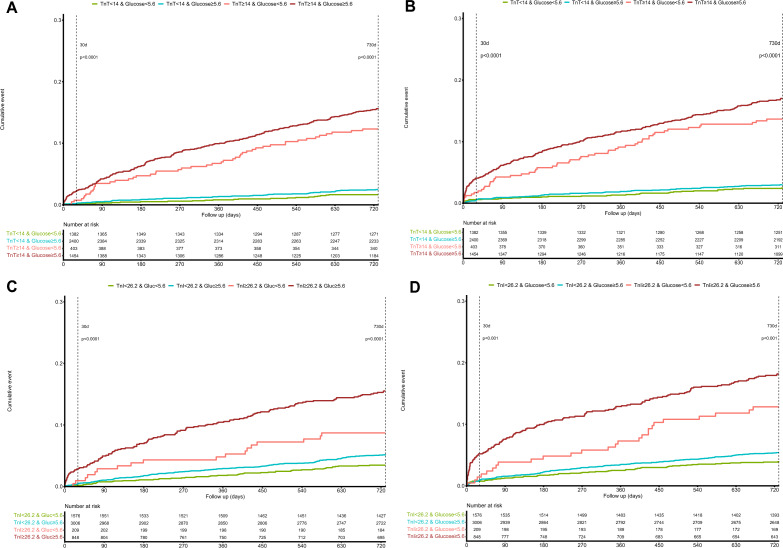
Kaplan Meier cumulative curves showing: (**A,C**) short-term and long-term survival of patients classified according to their hs-cTn and glucose concentrations; (**B,D**) short-term and long-term occurrence of the composite outcome cardiovascular death or AMI in patients classified according to their hs-cTn and glucose concentrations.

Patients with hs-cTn concentrations above the 99th percentile and glucose values ≥ 5.6 mmol/L showed a worse prognosis for both 30-day and 730-day outcomes (Fig. 3, Supplementary Results).

**Figure 4:**
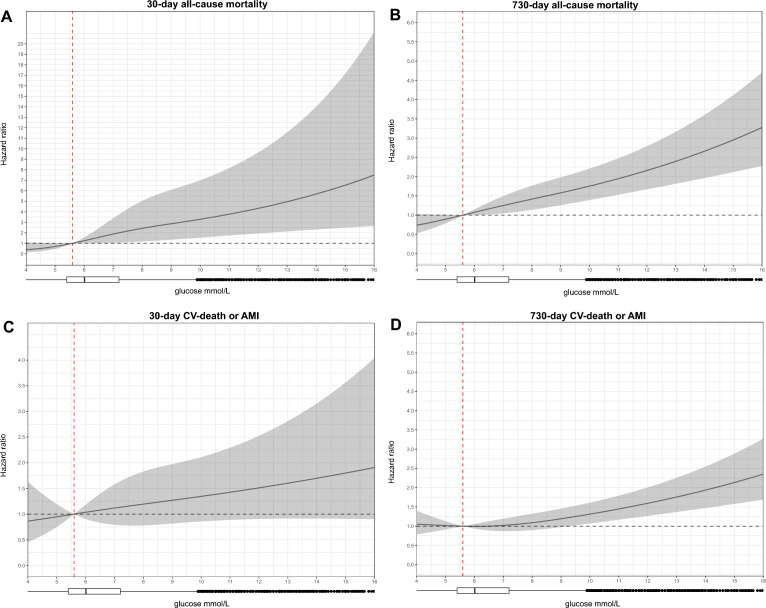
Dose–response plots showing the continuous non-linear association of glucose with: (**A**) overall 30-day mortality, (**B**) overall 730-day mortality, (**C**) 30-day cardiovascular death or AMI, (**D**) 730-day cardiovascular death or AMI. The graphs show the hazard ratio (HR) for glucose adjusted for: (**A**) age, GFR, diabetes mellitus, cancer; (**B–D**) age, gender, BMI, hypercholesterolemia, hypertension, active smoking status, diabetes mellitus, cancer and GFR. The shaded area represents the upper and lower limits of the 95% confidence interval. The horizontal dashed line represents an HR of 1, the vertical dashed line represents our glucose reference value (5.6 mmol/L). The box plots show the sample distribution. All 4 plots show 98% of the sample size, as extreme values would prevent good visualization, however, all data were used for the analysis and to create the graphs. *CV-death* cardiovascular death, *AMI* acute myocardial infarction.

Multivariable cox regression showed a non-linear association between glucose and 30-day and 2-year all-cause mortality (Fig. 4A,B). The figure shows the hazard ratio (HR) for each glucose value compared to the reference value (5.6 mmol/L). A statistically significant, non-linear association between glucose and 30-day mortality was observed for glucose values from 6.5 mmol/L onwards (Fig. 4A). A similar effect was observed for 2-year all-cause mortality (Fig. 4B).

In contrast to 30-day all-cause mortality, there was no association between glucose and the composite outcome of cardiovascular death or AMI at 30 days (Fig. 4C). For the 2-year outcome, a glucose value above 9.3 mmol/L was associated with a higher risk of cardiovascular death or AMI when compared to the reference value of 5.6 mmol/L (Fig. 4D). Previous history of diabetes was not an effect modifier for the association between glucose and the endpoints (likelihood ratio test P value = 0.79 for all-cause mortality and 0.51 for CV death or AMI) (Supplementary Fig. S5A,B).

## Discussion

This was a secondary analysis from a large, prospective, international diagnostic study using central adjudication by two independent cardiologists and long-term follow-up. We evaluated the clinical performance of a dual-marker approach combining glucose, a universally available inexpensive biochemical signature of diabetes mellitus and the physiological stress response to AMI, with either hs-cTnT or hs-cTnI in the early diagnosis of NSTEMI. We report six major findings.

First, glucose concentrations ≥ 5.6 mmol/L at ED presentation was present in more than two-thirds and identified patients at higher risk of AMI, as they were older, more often male, had a more than 4-times higher prevalence of diabetes mellitus, and a higher prevalence of known CAD versus patients with lower glucose values (< 5.6 mmol/L). Second, however, when analyzed as a continuous variable, glucose had only modest diagnostic accuracy for NSTEMI (AUC 0.63). In addition, the combination of glucose with either hs-TnT or hs-cTnI failed to further improve the diagnostic accuracy for NSTEMI already provided by hs-cTnT or hs-cTnI alone. Third, when investigating different dual-marker approaches to rule-out NSTEMI using only concentrations obtained at ED presentation (0 h blood draw) in patients presenting to the ED more than 3 h after chest pain onset, the target population for immediate rule-out according to current ESC guidelines, adding different glucose cut-offs to the ESC recommendations for hs-cTnT (< 5 ng/L) or hs-cTnI (< 4 ng/L) did not increase the sensitivity or NPV, but decreased the percentage of patients eligible for rule-out. Similarly, glucose did not improve, but deteriorate the rule-in process when assessed in all-comers. Fourth, when restricting the analysis to early presenters (patients presenting to the ED 3 h or less after onset of symptoms), a population for whom current guidelines do not recommend direct rule-out based on the 0 h blood draws, the dual-marker approach (glucose < 7.0 mmol/L and hs-cTnT < 5/hs-cTnI < 4 ng/L) provided very high and comparable sensitivity and rule-out efficacy to slightly lower hs-cTn concentrations (hs-cTnT < 4, hs-cTnI < 3 ng/L) alone. Fifth, adding glucose concentrations (< 5.6 mmol/L and ≥ 11.1 mmol/L) as triage criteria to the current ESC hs-cTn-0/1 h-algorithms overall did not increase its clinical performance for early rule-out and rule-in of NSTEMI. Sixth, multivariable cox regression showed a statistically significant association between glucose and 30-day all-cause mortality, 730-day all-cause mortality, and the composite of 730-day CV-mortality or future AMI. History of diabetes was not an effect modifier for the association between glucose and the prognostic endpoints.

These findings extend and corroborate prior studies aiming to further improve the early diagnosis of NSTEMI^29^, particularly those of the pilot data on evaluating the dual-biomarker approach combining glucose and hs-cTn assays^9,10,19^. At a time when still conventional cTn assays were used clinically at their institutions and the resulting “troponin-blind” interval was still very long, resulting in a major sensitivity deficit at ED presentation for the early diagnosis of AMI, research groups from France and Italy were the first to propose a possible diagnostic role for glucose^12,21^. This concept was further strengthened by studies from Australia, New Zealand, and Canada. Example, among 1412 patients presenting with acute chest discomfort to two EDs in Australia and New Zealand, of which 182 (12.9%) had index AMI, the dual-biomarker approach combining glucose and hs-cTnI had 100% sensitivity for index AMI, and identified 25.2% of patients as eligible for rule-out^9–11^. The current analysis differs in five important methodological details from these important pilot studies: first, it included central adjudication by two independent cardiologists using serial measurements of hs-cTn. This helped prevent misdiagnosis of small NSTEMIs that might have occurred in prior studies due to the suboptimal sensitivity of their reference standard. Second, predefined categories for the central adjudication in this study included “unknown cause” of acute chest discomfort. This reflects the clinical reality that in a small percentage of patients, the clinical work-up is either incomplete and does not include coronary angiography e.g.in frail elderly patients, or provides equivocal findings so that even experienced cardiologists adjudicating the event cannot exclude or confirm AMI with high enough certainty. The exclusion of patients with adjudicated “unknown cause” and at least one elevated hs-cTn concentration helped to prevent misdiagnosis of AMI. Third, it evaluated the combination of glucose with both hs-cTnT and hs-cTnI; This helped to further increase the generalizability of our findings. Fourth, it applied unbiased analysis of the diagnostic accuracy of glucose and hs-cTn evaluating them as quantitative variables. This helped prevent spurious positive findings for the dual-marker approach created by using suboptimal cut-offs for the single marker reference standard. Fifth, the very large and representative international multicenter study population helped to again further increase the generalizability of our findings.

Overall, the findings regarding the possible incremental value of glucose for the early diagnosis of AMI are remarkable similar to that of copeptin, which is secreted in an equimolar amount to arginine vasopressin, and has a central role in body fluid homeostasis, but also is rapidly upregulated as part of the systemic stress response^19,30–32^. The clinical introduction of hs-cTn-based rapid triage algorithms has massively reduced the sensitivity deficit of conventional cTn assays. In parallel, it reduced the diagnostic opportunity for an unspecific biomarker^33^. Our data confirms the high safety and efficacy of the ESC 0/1 h-algorithm (class I recommendation in the current ESC 2020 guidelines^2^) and shows that glucose has no additional diagnostic value for rule out of NSTEMI in patients presenting > 3 h after onset of symptoms.

Noteworthy, our findings regarding the possible value of glucose in combination with hs-cTn in early presenters highlight the possible clinical value of glucose in a dual marker approach for rule out of NSTEMI in addition to established hs-cTn single measurement rule-out concentrations. A glucose value < 7 mmol/L in combination with hs-cTnT < 5 ng/L or hs-cTnI < 4 ng/L achieved a very high safety for rule-out (sensitivity and NPV > 99%), and overall comparable rule-out performance to that of slightly lower hs-cTn concentrations (cTnT/I < 4/3 ng/L) alone, and possibly even higher efficacy. External validation of these findings is warranted prior to implementing them clinically. Furthermore, these findings should be corroborated with other commercially available hs-cTnI assays to assess whether the dual-marker strategy yields similar results to those obtained with Architect hs-cTnI assay.

Hyperglycemia in cardiac emergencies has been consistently shown to be associated with a higher risk of death and/or MACE^8^. Our data supports the current scientific consensus and is in harmony with several analyses that have shown an association between glucose concentration at presentation and both short-term and long-term outcomes^10,34–37^.

Some limitations merit consideration when interpreting these findings. First, this study was conducted in patients presenting with acute chest discomfort/pain to the ED. Further studies are required to evaluate the possible diagnostic role of glucose in patients with a lower pre-test probability for NSTEMI, e.g., in a general practitioner setting. Second, we cannot generalize our findings to patients with terminal kidney disease requiring dialysis, since they were excluded from this study. Third, information on the fasting status in the patients included in this study was unknown. These results should be interpreted in the context of a pragmatic assessment of random glucose concentrations at presentation. Future studies need to test the hypothesis that adjusting glucose concentration for the time since last food intake would further increase the prognostic accuracy of glucose concentration.

In conclusion, the use of a dual-biomarker approach adding glucose to hs-cTnT or hs-cTnI did not improve the diagnostic accuracy for the rapid rule-out or rule-in of NSTEMI in patients presenting with acute chest discomfort to the ED. However, the possible incremental value of glucose in combination with hs-cTn in patients presenting very early after onset of symptoms warrants further study. Glucose was associated with short- and long-term mortality, irrespective of the presence or absence of diabetes mellitus.

### Supplementary Information

Supplementary Information.

## Data Availability

The data, code, and study material that support the findings of this study are available from the corresponding author on reasonable request.
