# Supplementary material for: Combining glucose and high-sensitivity cardiac troponin in the early diagnosis of acute myocardial infarction
Source: Sci Rep. 2023 Sep 5;13:14598. doi: 10.1038/s41598-023-37093-1 (PMC10480296; doi:10.1038/s41598-023-37093-1)
Supplement: Supplementary file 1 — Supplementary Information. [file 41598_2023_37093_MOESM1_ESM.pdf]

# Combining Glucose and High-Sensitivity Cardiac Troponin T and I in the Early Diagnosis of Acute Myocardial Infarction

# SUPPLEMENTARY MATERIAL

Ana Yufera-Sanchez, MD[1,2**]; Pedro Lopez-Ayala, MD[1,2**]; Thomas Nestelberger, MD[1,2,] Karin Wildi, MD[1,2,3]; Jasper Boeddinghaus, MD[1,2,4]; Luca Koechlin, MD[1,2,6]; Maria Rubini Gimenez, MD[1,5]; Hüseyin Sakiz, MS[1,2]; Paolo Bima, MD[1,2,7]; Oscar Miro, MD[2,8]; F. Javier Martín-Sánchez, MD[2,9]; Michael Christ, MD[10]; Dagmar I. Keller, MD[11]; Danielle M. Gualandro, MD, PhD[1,2]; Damian Kawecki, MD[12]; Katharina Rentsch, PhD[13]; Andreas Buser, MD[14]; Christian Mueller, MD[1,2*] on behalf of the APACE Investigators[#]

[1]Department of Cardiology, University Heart Center Basel, and Cardiovascular Research Institute Basel (CRIB), University Hospital Basel, University of Basel, Switzerland; [2]GREAT network; [3] Department of Intensive Care, University Hospital Basel, University of Basel, Switzerland; [4]Department of Cardiology, Royal Infirmary of Edinburgh; [5]Cardiology Department, Heart Center Leipzig, Leipzig, Germany; [6]Department of Cardiac Surgery, University Hospital Basel, University of Basel, Switzerland; [7]Department of Medical Sciences, University of Torino, Italy; [8]Emergency Department, Hospital Clinic, Barcelona, Catalonia, Spain; [9]Emergency Department, Hospital Clínico San Carlos, Madrid, Spain; [10]Department of Emergency Medicine, Luzerner Kantonsspital, Luzern, Switzerland; [11]Emergency Department, University Hospital Zurich, Zurich, Switzerland; [12]2nd Department of Cardiology, School of Medicine in Zabrze, Medical University of Sielsia, Katowice, Poland; [13]Laboratory Medicine, University Hospital Basel, University of Basel, Switzerland [14]Blood Transfusion Centre, Swiss Red Cross, Basel, Switzerland and Department of Hematology, University Hospital Basel, University of Basel, Switzerland

***both authors contributed equally and should be considered first author*

Trial Registration: **ClinicalTrials.gov number, NCT00470587**

## Supplementary Methods

1. **Clinical assessment**
2. **Blood sampling and laboratory methods**
3. **Follow-up**
4. **Statistical analysis**

## Supplementary Results

1. Prognostic performance of hs-cTn and glucose.

## Supplementary Tables

1. **Supplementary Table S1 –** STROBE Statement—checklist of items that should be included in reports of observational studies
2. **Supplemental Table S2 –** Baseline Characteristics of patients with and without NSTEMI
3. **Supplementary Table S3 –** Diagnostic performance of different glucose cut-offs for rule-out of NSTEMI in all commers
4. **Supplementary Table S4**
   - **Table S4A –** Diagnostic accuracy of hs-cTnT alone and its combination with glucose for different subgroups.
   - **Table S4B –** Diagnostic accuracy of hs-cTnI alone and its combination with glucose for different subgroups.
5. **Supplementary Tables S5A and B –** Sensitivity analysis for the diagnosis of NSTEMI Type 1 and NSTEMI Type 2, respectively, with hs-cTn alone and its combination with glucose.

## Supplementary Figures

1. **Supplementary Figure S1 –** Patient Flowchart.

2. **Supplementary Figure S2 - ESC** 0/1h Algorithm and concept outlining on how the 0h glucose concentration could be used in combination with it.

    - **Figure S2A)** ESC 0/1h Algorithm hs-cTnT Elecsys and its combination with glucose.
    - **Figure S2B)** ESC 0/1h Algorithm hs-cTnI Abott and its combination with glucose.

3. **Supplementary Figure S3** – Boxplots showing concentrations of glucose at admission according to the adjudicated final diagnosis.

4. **Supplementary Figure S4 -** Diagnostic accuracy of hs-cTnT (**4A**), hs-cTnI (**4B**) and their combination with glucose at presentation in the ED for the diagnosis of NSTEMI.

5. **Supplementary Figure S5** – Dose-response plots for prognostic outcomes for patients with and without diabetes
    - **Figure S5A)** 730-days all-cause mortality.
    - **Figure S5B)** 730-days cardiovascular death or AMI.

## Supplementary Methods

### Clinical Assessment

Routine clinical assessment and patient management has been described in detail previously [1]. The estimated glomerular filtration rate (eGFR) was determined using the chronic kidney disease epidemiology collaboration (CKD-MDRD) formula [2].

### Blood sampling and laboratory methods

Glucose levels were measured from routine blood samples obtained at ED presentation on the clinical chemistry platform of each participating hospital. Blood sampling and methods for the determination of hs-cTnT (Elecsys) and hs-cTnI (Architect) concentrations have been previously reported [1,3–5].

### Follow-up

Patients were interviewed by telephone or in written form after 3, 12 and 24 months. Contact was established with the patient and the family physician. Information regarding mortality was also obtained from the national death registries, the electronic medical record of the hospital or family physicians records.

### Statistical analysis

To evaluate whether the presence of diabetes could be an effect modifier of glucose, an interaction between glucose and diabetes was fitted. Considering the low number of events in each subgroup (diabetic/non-diabetic patients) for 30-days (short term) and to avoid overfitting, the interaction was only assessed for 2-year outcomes. To compute the hazard ratios (Y-axis) the reference was a glucose value of 5.6 mmol/L and non-diabetic. The multivariable model had the same covariates used for the main analysis. The only

difference was the interaction. Hence, a likelihood ratio test for nested models was used for evaluating the interaction between glucose and diabetes. In addition, effect modification of diabetes was assessed visually with dose-response plots.

Both prognostic outcomes (all-cause mortality and the composite of cardiovascular death and AMI) were plotted in Kaplan–Meier curves for 30 days and 730-days follow-up time according to hs-cTn and glucose baseline concentrations. The log rank test was used to assess differences between groups:

- **Group 1:** hs-cTn concentrations below the 99$^{th}$ percentile and glucose concentrations below 5.6 mmol/L.
- **Group 2:** hs-cTn concentrations below the 99$^{th}$ percentile and glucose concentrations ≥ 5.6mmol/L.
- **Group 3:** hs-cTn concentrations over the 99$^{th}$ percentile and glucose concentrations below 5.6mmol/L.
- **Group 4:** hs-cTn concentrations over the 99$^{th}$ percentile and glucose concentrations ≥ 5.6mmol/L.

## Supplementary Results

### Prognostic performance of hs-cTn and glucose

The median duration of follow-up was 1401 days (IQR 764-2723) with 43 (0.8%) deaths occurring within 30 days and 353 (6.3%) within 2 years.

Cumulative 30-days survival rates were 99.0% (14 events) in the group of patients with hs-cTnT values below the 99$^{th}$ percentile and glucose below 5.6 mmol/L **(Group 1)**; 99.7% (8 events) in patients with hs-cTnT below 14ng/L and glucose ≥5.6mmol/L **(Group 2);** 99.3% (3 events) in patients with hs-cTnT over the 99$^{th}$ percentile and glucose below 5.6mmol/L **(Group 3)** and 97.5% (36 events, log rank., p<0.001) in patients with hs-cTnT over the 99$^{th}$ percentile and glucose ≥5.6mmol/L **(Group 4) (Figure 3A).** At 2 years, cumulative survival rates were 92% (111 events), 93% (167 events), 84.4% (63 events), 81.4% (270 events) respectively (log-rank, p<0.001).

Cumulative 30-days survival rates were 99.1% (18 events) in the group of patients with hs-cTnI values below the 99$^{th}$ percentile and glucose below 5.6 mmol/L **(Group 1)**; 99.4% (19 events) in patients with hs-cTnI below 26.2ng/L and glucose ≥5.6mmol/L **(Group 2);** 98.5% (3 events) in patients with hs-cTnI over the 99$^{th}$ percentile and glucose below 5.6mmol/L **(Group 3)** and 97.0% (25 events, log rank., p<0.001) in patients with hs-cTnI over the 99$^{th}$ percentile and glucose ≥5.6mmol/L **(Group 4) (Figure 3C).** At 2 years, cumulative survival rates were 90.6% (149 events), 90.5% (286 events), 87.7% (25 events), 81.9% (151 events) respectively (log-rank, p<0.001).

Similar findings were observed for the composite outcome of cardiovascular death and AMI excluding index event at 30 days and 2 years for both hs-cTnT and hs-cTnI **(Figure 3B, 3**

## Supplementary Tables

**Table 1:** STROBE Statement—checklist of items that should be included in reports of observational studies [6].

| | Item No | Recommendation | Page |
|---|---|---|---|
| **Title and abstract** | 1 | (*a*) Indicate the study's design with a commonly used term in the title or the abstract | 2 |
| | | (*b*) Provide in the abstract an informative and balanced summary of what was done and what was found | 2 |
| **Introduction** | | | |
| Background/rationale | 2 | Explain the scientific background and rationale for the investigation being reported | 3 |
| Objectives | 3 | State specific objectives, including any prespecified hypotheses | 3 |
| **Methods** | | | |
| Study design | 4 | Present key elements of study design early in the paper | 4-6 |
| Setting | 5 | Describe the setting, locations, and relevant dates, including periods of recruitment, exposure, follow-up, and data collection | 4-6 + S. 4 |
| Participants | 6 | (*a*) *Cohort study*—Give the eligibility criteria, and the sources and methods of selection of participants. Describe methods of follow-up<br>*Case-control study*—Give the eligibility criteria, and the sources and methods of case ascertainment and control selection. Give the rationale for the choice of cases and controls<br>*Cross-sectional study*—Give the eligibility criteria, and the sources and methods of selection of participants | 4 + S.4 |
| | | (*b*) *Cohort study*—For matched studies, give matching criteria and number of exposed and unexposed<br>*Case-control study*—For matched studies, give matching criteria and the number of controls per case | N.A. |
| Variables | 7 | Clearly define all outcomes, exposures, predictors, potential confounders, and effect modifiers. Give diagnostic criteria, if applicable | 4-8 + S.4 |
| Data sources/ measurement | 8* | For each variable of interest, give sources of data and details of methods of assessment (measurement). Describe comparability of assessment methods if there is more than one group | 6-8 |
| Bias | 9 | Describe any efforts to address potential sources of bias | 4-8 |

| | | | |
|---|---|---|---|
| Study size | 10 | Explain how the study size was arrived at | Figure S1 |
| Quantitative variables | 11 | Explain how quantitative variables were handled in the analyses. If applicable, describe which groupings were chosen and why | 7-8 |
| Statistical methods | 12 | (*a*) Describe all statistical methods, including those used to control for confounding | 6-8 |
| | | (*b*) Describe any methods used to examine subgroups and interactions | 6-8 |
| | | (*c*) Explain how missing data were addressed | N.A. |
| | | (*d*) *Cohort study*—If applicable, explain how loss to follow-up was addressed<br>*Case-control study*—If applicable, explain how matching of cases and controls was addressed<br>*Cross-sectional study*—If applicable, describe analytical methods taking account of sampling strategy | N.A. |
| | | (*e*) Describe any sensitivity analyses | 6-8 |

| | | | |
|---|---|---|---|
| **Results** | | | |
| Participants | 13* | (a) Report numbers of individuals at each stage of study—eg numbers potentially eligible, examined for eligibility, confirmed eligible, included in the study, completing follow-up, and analysed | Fig. S1 |
| | | (b) Give reasons for non-participation at each stage | N.A. |
| | | (c) Consider use of a flow diagram | Fig. S1 |
| Descriptive data | 14* | (a) Give characteristics of study participants (eg demographic, clinical, social) and information on exposures and potential confounders | Table 1 & Table S2 |
| | | (b) Indicate number of participants with missing data for each variable of interest | Fig. S1 |
| | | (c) *Cohort study*—Summarise follow-up time (eg, average and total amount) | S. 4 |
| Outcome data | 15* | *Cohort study*—Report numbers of outcome events or summary measures over time | S. 6 |
| | | *Case-control study*—Report numbers in each exposure category, or summary measures of exposure | N.A. |
| | | *Cross-sectional study*—Report numbers of outcome events or summary measures | N.A. |
| Main results | 16 | (*a*) Give unadjusted estimates and, if applicable, confounder-adjusted estimates and their precision (eg, 95% confidence interval). Make clear which confounders were adjusted for and why they were included | 10-14, Fig. 3-4 Fig. S5 |
| | | (*b*) Report category boundaries when continuous variables were categorized | 5-7 |
| | | (*c*) If relevant, consider translating estimates of relative risk into absolute risk for a meaningful time period | N.A. |

| | | | |
|---|---|---|---|
| Other analyses | 17 | Report other analyses done—eg analyses of subgroups and interactions, and sensitivity analyses | 11-14 + S. 6 + Table S4-5 + Figure S2-5 |
| **Discussion** | | | |
| Key results | 18 | Summarise key results with reference to study objectives | 15-16 |
| Limitations | 19 | Discuss limitations of the study, taking into account sources of potential bias or imprecision. Discuss both direction and magnitude of any potential bias | 17-19 |
| Interpretation | 20 | Give a cautious overall interpretation of results considering objectives, limitations, multiplicity of analyses, results from similar studies, and other relevant evidence | 16-18 |
| Generalisability | 21 | Discuss the generalisability (external validity) of the study results | 16-18 |
| **Other information** | | | |
| Funding | 22 | Give the source of funding and the role of the funders for the present study and, if applicable, for the original study on which the present article is based | 19 |

*Give information separately for cases and controls in case-control studies and, if applicable, for exposed and unexposed groups in cohort and cross-sectional studies.

**Supplementary Table 2.**

Baseline Characteristics of patients with and without NSTEMI

| | Total cohort n=5639 | NO NSTEMI n=4588 (81.4%) | NSTEMI n=1051 (18.6 %) | p-values |
|---|---|---|---|---|
| **Descriptive factors** | | | | |
| Female gender - n° (%) | 1889 (33.5) | 1590 (34.7) | 299 (28.4) | <0.001 |
| Age, median (IQR) | 61 (49-74) | 59 (47-72) | 70 (59-80) | <0.001 |
| BMI, median (IQR) | 26.5 (23.9-29.7) | 26.4 (23.8-29.7) | 26.6 (24.2-29.4) | 0.359 |
| Chest pain onset, median (IQR) | 5 (2-12) | 5 (2-12.5) | 5 (2-11) | 0.615 |
| Early presenters (CPO<=3h) - n° (%) | 2093 (37.1%) | 1710 (37.3) | 383 (36.4) | 0.616 |
| **Vital parameters, median IQR** | | | | |
| Systolic BP - mmHg | 140 (125-156) | 139 (125-155) | 141 (125-160) | 0.03 |
| Diastolic BP - mmHg | 80 (71-90) | 81 (71-90) | 80 (70-91) | 0.207 |
| Heart rate - bpm | 76 (66-89) | 76 (66-88) | 78 (68-92) | <0.001 |
| **Risks factors – n° (%)** | | | | |
| Hypertension | 3378 (59.9) | 2564 (55.9) | 814 (77.5) | <0.001 |
| Hypercholesterolemia | 2672 (47.4) | 2018 (44.0) | 654 (62.2) | <0.001 |
| Diabetes Mellitus | 982 (17.3) | 696 (15.2) | 286 (27.2) | <0.001 |
| Active smoker | 1397 (24.8) | 1153 (25.1) | 244 (23.2) | 0.195 |
| **Patients's history – n° (%)** | | | | |
| Coronary artery disease | 1834 (32.5) | 1365 (29.8) | 469 (44.6) | <0.001 |
| Prior AMI | 1310 (23.2) | 965 (21.0) | 345 (32.8) | <0.001 |
| Prior revascularization | 1538 (27.3) | 1163 (25.3) | 375 (35.7) | <0.001 |
| Prior stroke | 297 (5.3) | 203 (4.4) | 94 (8.9) | <0.001 |
| Renal impairment | 298 (5.3) | 349 (7.6) | 192 (18.3) | <0.001 |
| **Medication at presentation – n° (%)** | | | | |
| Platelet inhibitor | 2155 (38.2) | 1623 (35.4) | 532 (50.6) | <0.001 |
| Beta-blocker | 1903 (33.7) | 1451 (31.6) | 452 (43.0) | <0.001 |
| ACE-Inhibitor and ARB | 2220 (39.4) | 1660 (36.2) | 560 (53.3) | <0.001 |
| Statin | 1977 (35.1) | 1512(33.0) | 465 (44.2) | <0.001 |
| Calcium antagonists | 852 (15.1) | 636 (13.9) | 216 (20.6) | <0.001 |
| Nitrates | 543 (9.6) | 380 (8.3) | 163 (15.5) | <0.001 |
| Oral antidiabetics | 538 (9.5) | 382 (8.4) | 156 (15.0) | <0.001 |
| Insulin | 310 (5.5) | 219 (4.8) | 91 (8.8) | <0.001 |
| **Blood test at admission, median (IQR)** | | | | |
| Glucose - mmol/L | 6.00 (5.40-7.20) | 5.9 (5.3- 6.9) | 6.6 (5.7-8.7) | <0.001 |
| Hemoglobin - g/L | 143 (132-153) | 143 (133-153) | 141 (126-153) | <0.001 |
| GFR MDRD - ml/min/1.73m2 | 84.0 (68.4-100.0) | 85.8 (70.9-101.6) | 73.8 (58.2-92.8) | <0.001 |
| High sensitivity Troponin T - ng/L | 8.0 (4.0-19.0) | 6.0 (4.0-12.0) | 49.0 (24.0-122.0) | <0.001 |
| High sensitivity Troponin I - ng/L | 4.1 (2.0-15.0) | 3.1 (2.0-6.9) | 77.3 (20.9-428.8) | <0.001 |
| **Glucose (≥5.6 mmol/L)** | 3854 (68.3%) | 3008 (65.6) | 846 (80.5) | <0.001 |

Man-Whitney U test for continuous variables (not normal distributed), expressed in medians and interquartile range (IQR) and Chi-square test for categorical variables, expressed in numbers and percentages.

NSTEMI= non-ST elevation myocardial infarction, SBP= systolic blood pressure, CPO = chest pain onset, DBP= diastolic blood pressure, CAD= coronary artery disease, AMI= acute myocardial infarction, ACE-inhibitor= angiotensin-converting-enzyme inhibitor, ARB= angiotensin receptor blocker, GFR-MDRD= glomerular filtration rate-modification of diet in renal disease equation.

**Supplementary Table 3.** Diagnostic performance of different glucose cut-offs for rule-out of NSTEMI in all commers (n = 5639).

| | Sensitivity | NPV | Patients ruled out | False negative |
|---|---|---|---|---|
| **Glucose <5.6 (mmol/L)** | 80.5 (78.0-82.8) | 88.5 (87.0-89.9) | 1785 | 205 |
| **Glucose <6.1 (mmol/L)** | 63.7 (60.7-66.5) | 86.5 (85.2-87.8) | 2839 | 382 |
| **Glucose <7 (mmol/L)** | 43.0 (40.0-46.0) | 85.2 (84.1-86.3) | 4050 | 599 |

NPV = negative predictive value

**Supplementary Table 4A**. Diagnostic accuracy of hs-cTnT alone and its combination with glucose for different subgroups. Patients with a final adjudicated diagnosis based on hs-cTnT concentrations were considered for this analysis.

| Subgroups | | N° of patients | ROC AUC (95%CI) | p-value |
|---|---|---|---|---|
| **Early presenters (CPO ≤3 hours)** | Hs-cTnT alone | 2093 | 0.911 (0.897 - 0.926) | 0.588 |
| | Hs-cTnT and glucose | | 0.912 (0.898 - 0.927) | |
| **CPO > 3 hours** | Hs-cTnT alone | 3546 | 0.945 (0.937 - 0.953) | 0.984 |
| | Hs-cTnT and glucose | | 0.945 (0.937 - 0.953) | |
| **Intermediate troponin concentrations (≥ 5ng/L - <52ng/L)** | Hs-cTnT alone | 3427 | 0.851 (0.834 - 0.867) | 0.057 |
| | Hs-cTnT and glucose | | 0.853 (0.837 - 0.870) | |
| **Diabetic patients** | Hs-cTnT alone | 982 | 0.901 (0.882 - 0.920) | 0.462 |
| | Hs-cTnT and glucose | | 0.902 (0.883 - 0.922) | |
| **Non diabetic patients** | Hs-cTnT alone | 4657 | 0.933 (0.924 - 0.942) | 0. 120 |
| | Hs-cTnT and glucose | | 0.934 ( 0.926 - 0.943) | |

ROC = Receiver operating Curve, AUC = Are Under the Curve, CPO= chest pain onset.

**Supplementary Table 4B**. Diagnostic accuracy of hs-cTnI alone and its combination with glucose for different subgroups. Patients with a final adjudicated diagnosis based on hs-cTnI concentrations were considered for this analysis.

| Subgroups | | N° of patients | ROC AUC (95%CI) | p-value |
|---|---|---|---|---|
| **Early presenters (CPO ≤3 hours)** | Hs-cTnI alone | 1842 | 0.926 (0.912 - 0.939) | 0.523 |
| | Hs-cTnI and glucose | | 0.927 (0.914 - 0.941) | |
| **CPO > 3 hours** | Hs-cTnI alone | 3139 | 0.959 (0.953 - 0.966) | 0.337 |
| | Hs-cTnI and glucose | | 0.959 (0.952 - 0.966) | |
| **Intermediate troponin concentrations (≥ 4ng/L – <64ng/L)** | Hs-cTnI alone | 2018 | 0.829 (0.807 - 0.852) | 0.035 |
| | Hs-cTnI and glucose | | 0.835 (0.813 - 0.857) | |
| **Diabetic patients** | Hs-cTnI alone | 868 | 0.938 (0.922 – 0.954) | 0.535 |
| | Hs-cTnI and glucose | | 0.939 (0.923 – 0.955) | |
| **Non diabetic patients** | Hs-cTnI alone | 4113 | 0.945 (0.937 – 0.952) | 0. 244 |
| | Hs-cTnI and glucose | | 0.946 (0.939 – 0.954) | |

ROC = Receiver operating characteristic curve, AUC = Area Under the Curve, CPO= chest pain onset.

**Supplementary Table 5A:** Sensitivity analysis for the diagnosis of NSTEMI Type 1 and NSTEMI Type 2, respectively, with hs-cTnT alone and its combination with glucose.

| | | ROC AUC (95%CI) | p-value |
|---|---|---|---|
| **Outcome Type 1 MI** | Hs-cTnT alone | 0.925 (0.916 – 0.933) | 0.693 |
| | Hs-cTnT and glucose | 0.925 (0.917 – 0.934) | |
| **Outcome Type 2 MI** | Hs-cTnT alone | 0.813 (0.795 - 0.831) | 0.243 |
| | Hs-cTnT and glucose | 0.807 (0.789 - 0.826) | |

**Supplementary Table 5B:** Sensitivity analysis for the diagnosis of NSTEMI Type 1 and NSTEMI Type 2, respectively, with hs-cTnI alone and its combination with glucose.

| | | ROC AUC (95%CI) | p-value |
|---|---|---|---|
| **Outcome Type 1 MI** | Hs-cTnI alone | 0.928 (0.920 - 0.935) | 0.354 |
| | Hs-cTnI and glucose | 0.926 (0.918 - 0.934) | |
| **Outcome Type 2 MI** | Hs-cTnI alone | 0.828 (0.810 – 0.847) | 0.055 |
| | Hs-cTnI and glucose | 0.818 (0.800 – 0.837) | |

# Supplementary Figures

**Supplementary Figure S1.** Patient Flowchart in the APACE study from April 2006 to April 2018.

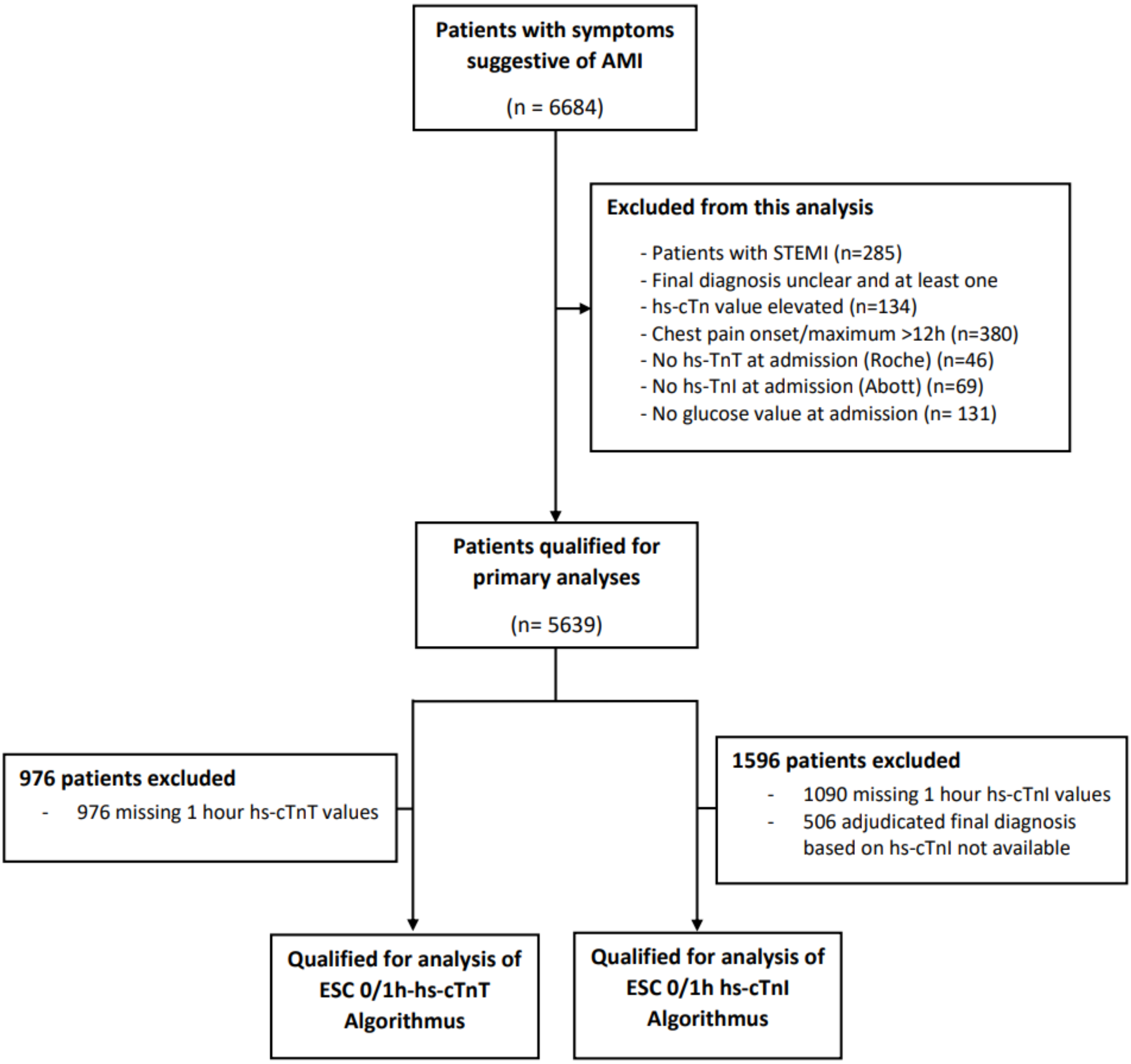


STEMI indicates ST elevation myocardial infarction; AMI, acute myocardial infarction; hs-cTnT, high-sensitivity cardiac troponin T; hs-cTnI, high-sensitivity cardiac troponin I and NSTEMI indicates non-ST elevation myocardial infarction.

**Supplementary Figure S2.** ESC 0/1h hs-cTn Algorithm and concept outlining on how the 0h glucose concentration could be used in combination with the ESC 0/1h-algorithm.

**2A**

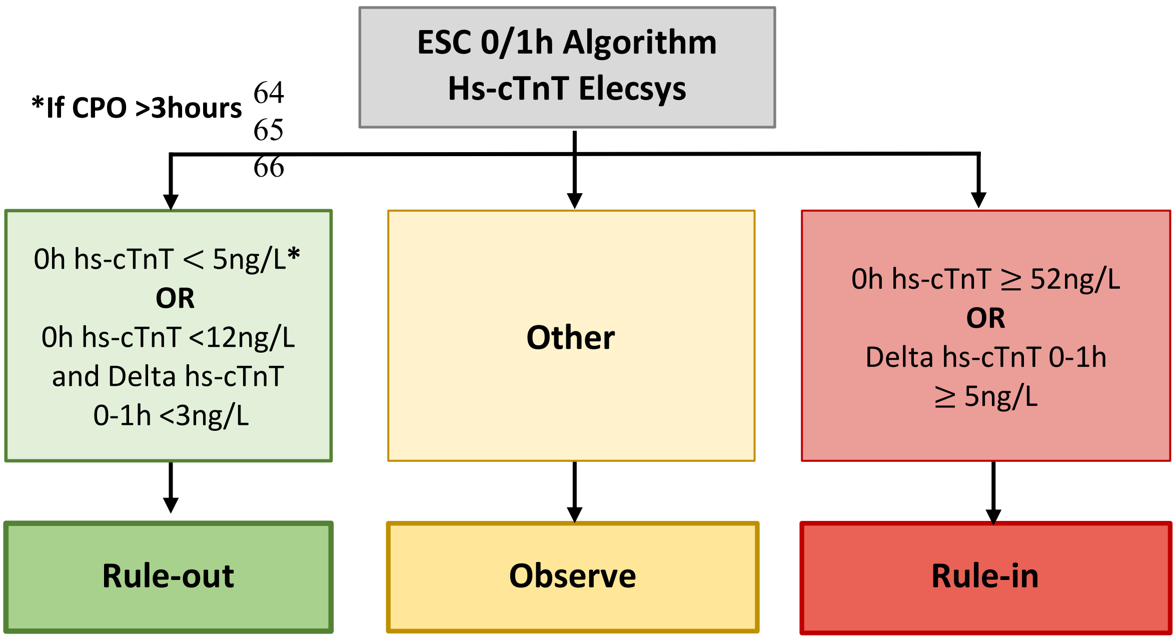


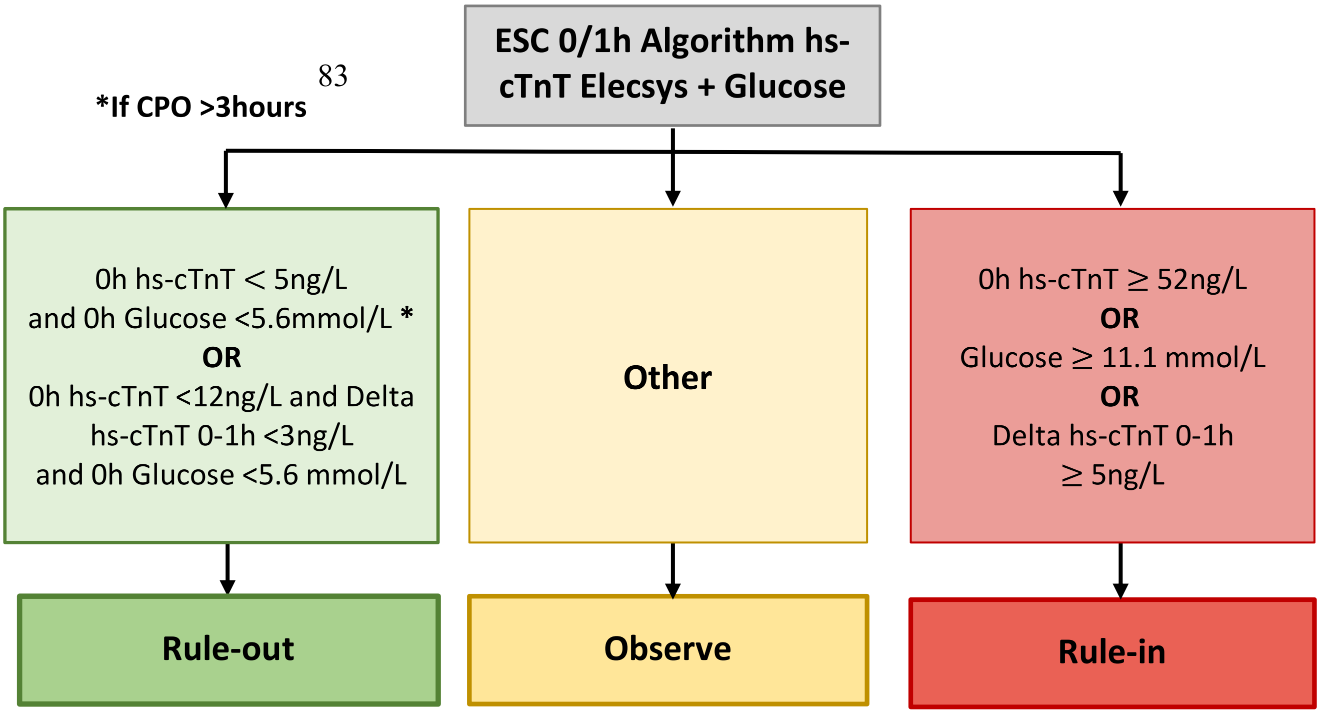

**2B**

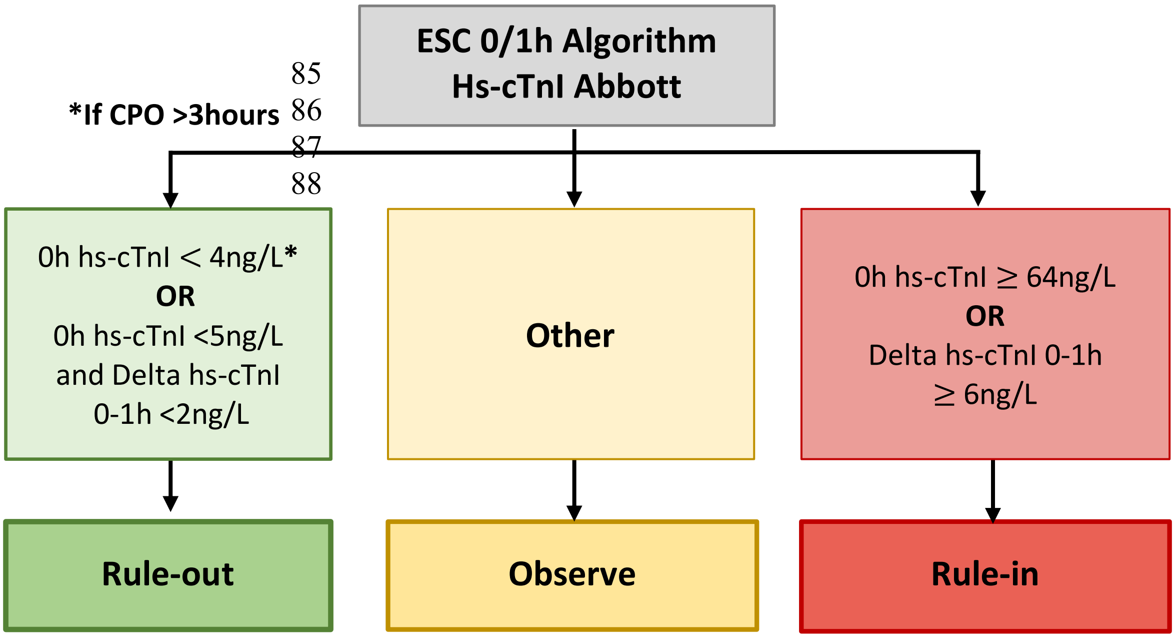


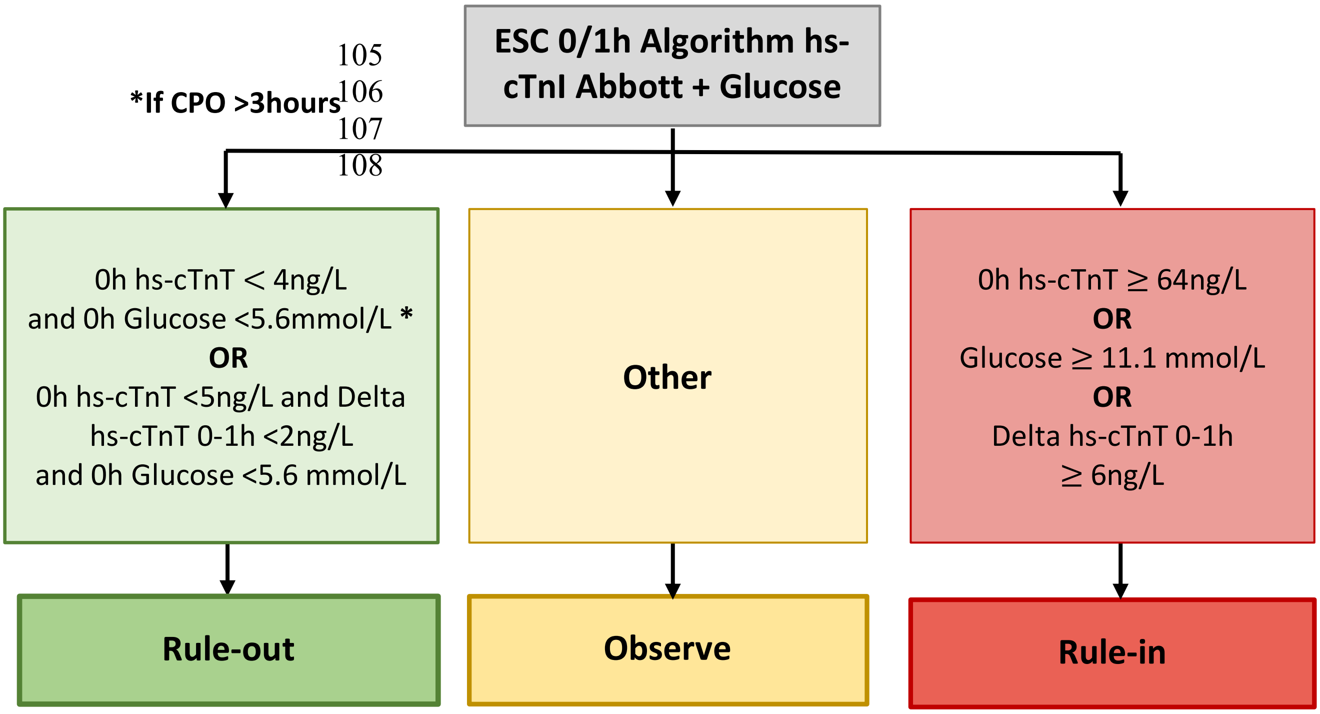


NSTEMI indicates non-ST-Elevation myocardial infarction; hs-cTnT indicates high sensitivity cardiac troponin T; hs-cTnI indicates high sensitivity cardiac troponin I.

**Supplementary Figure S3.** Boxplots showing concentrations of glucose at admission according to the adjudicated final diagnosis.

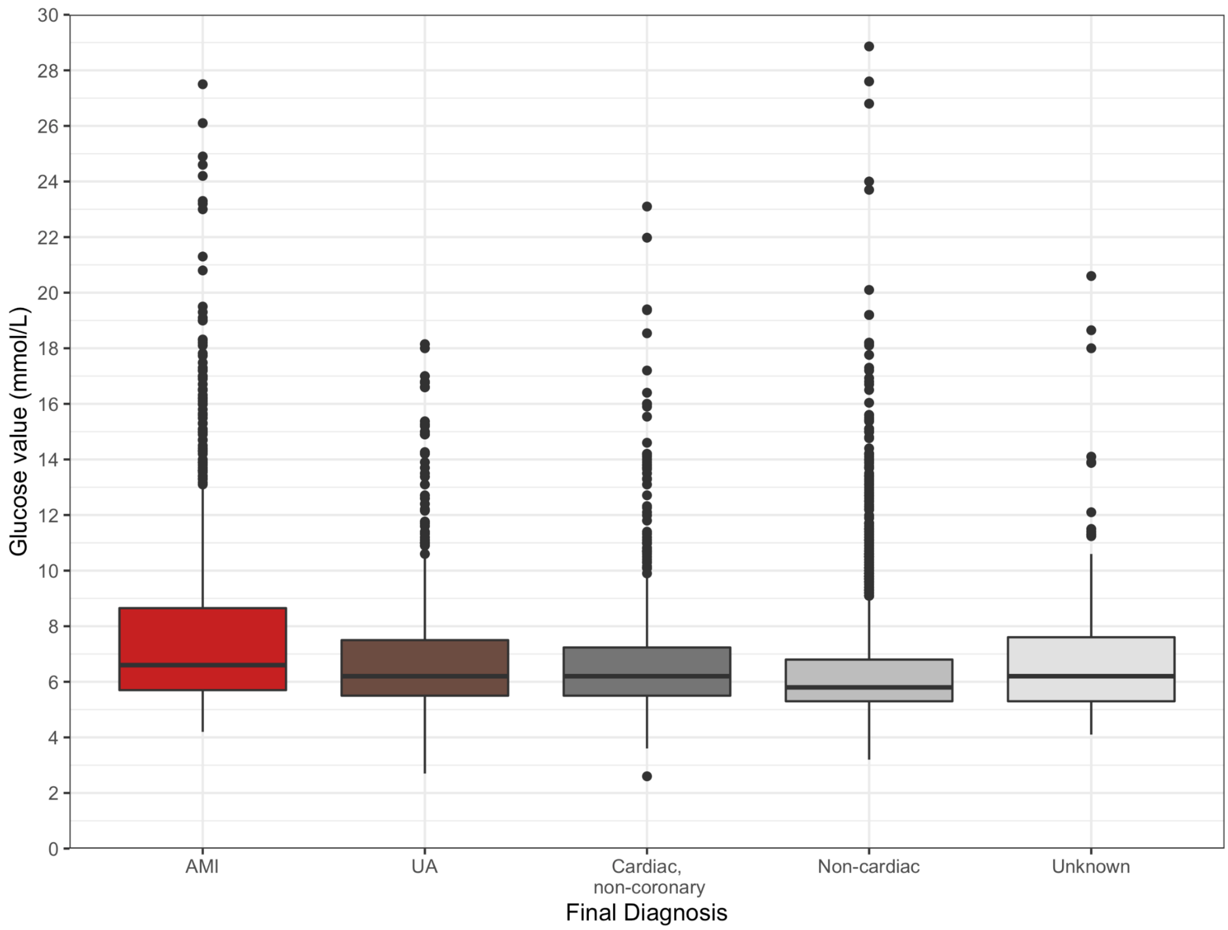


Boxes represent medians and interquartile ranges (IQRs). End of lines display the smallest and the largest non-outliers. Outliers are displayed by the dots. AMI indicates acute myocardial infarction; UA indicates unstable angina.

**Supplementary Figure S4.** Diagnostic accuracy of hs-cTnT (**2A**), hs-cTnI (**2B**) and their combination with glucose at presentation in the ED for the diagnosis of NSTEMI.

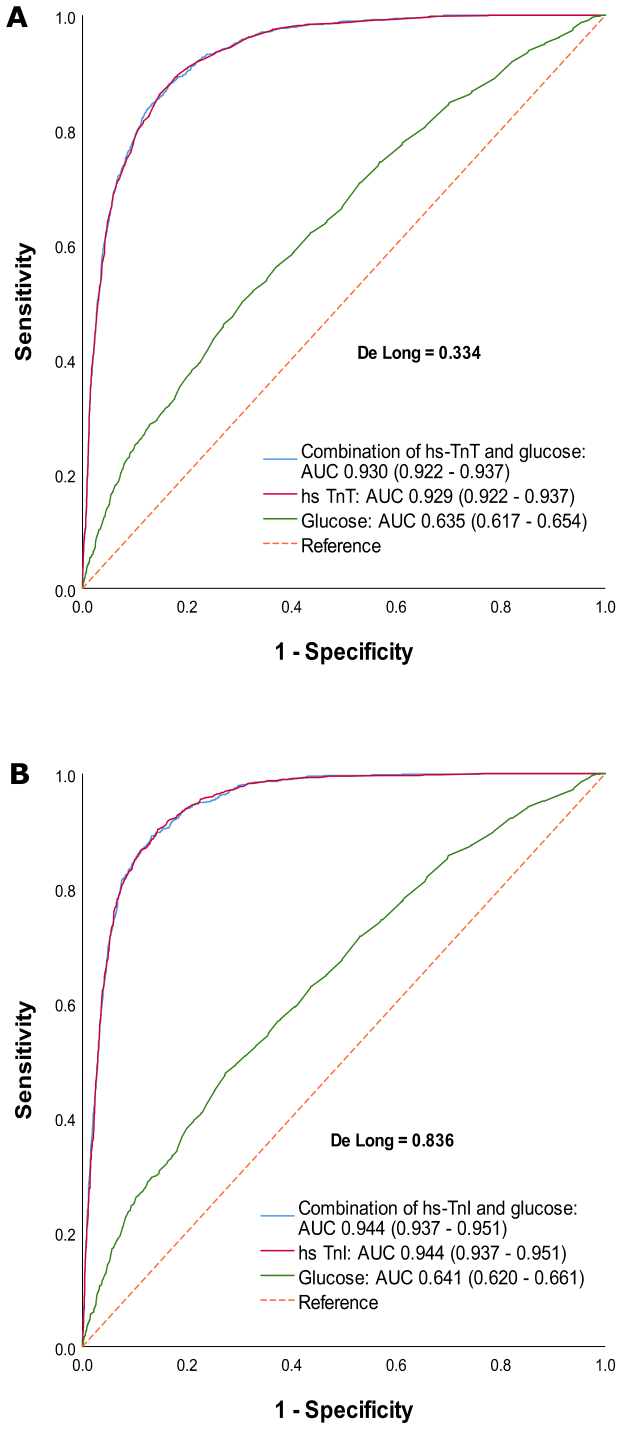


AUC denotes area under the receiver-operating-characteristic (ROC) curve, hs-cTnT denotes high sensitivity cardiac troponin T, hs-cTnI denotes high sensitivity cardiac troponin I.

**Supplementary Figure S5A and B** – Dose-response plots for patients with and without diabetes for **A)** 2-year all-cause mortality and **B)** 2 -year CV death or AMI.

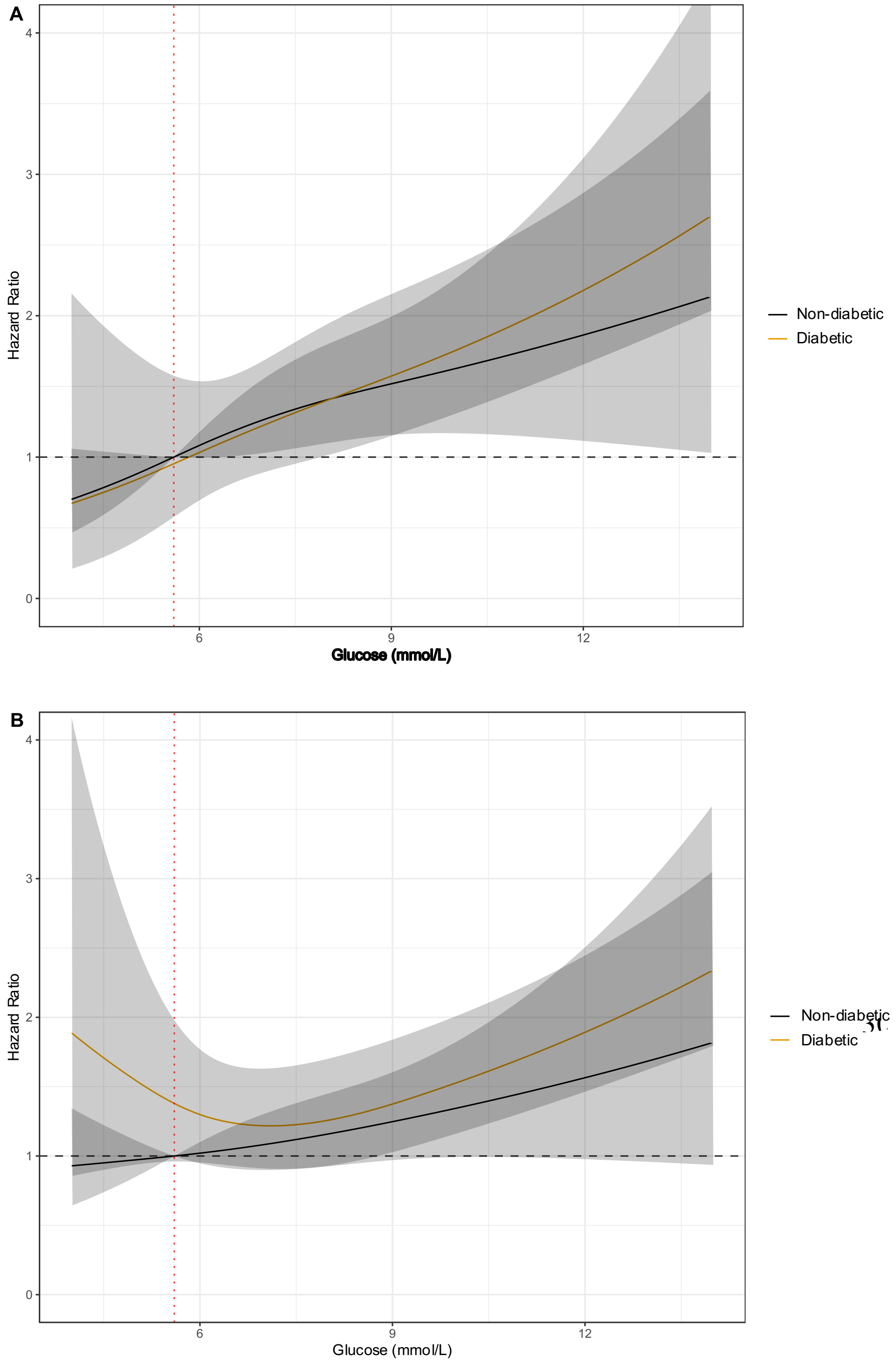

## References


1. Twerenbold, R. *et al.* Outcome of Applying the ESC 0/1-hour Algorithm in Patients With Suspected Myocardial Infarction. *J. Am. Coll. Cardiol.* **74**, 483–494 (2019).

2. Levey, A. S. *et al.* Using standardized serum creatinine values in the modification of diet in renal disease study equation for estimating glomerular filtration rate. *Ann. Intern. Med.* **145**, 247–254 (2006).

3. Boeddinghaus, J. *et al.* Early Diagnosis of Myocardial Infarction With Point-of-Care High-Sensitivity Cardiac Troponin I. *J. Am. Coll. Cardiol.* **75**, 1111–1124 (2020).

4. Nestelberger, T. *et al.* Direct comparison of high-sensitivity cardiac troponin T and I in the early differentiation of type 1 vs. type 2 myocardial infarction. *Eur. Hear. Journal. Acute Cardiovasc. Care* **11**, 62–74 (2022).

5. Lopez-Ayala, P. *et al.* Novel Criteria for the Observe-Zone of the ESC 0/1h-hs-cTnT Algorithm. *Circulation* 773–787 (2021).

6. von Elm, E. *et al.* The Strengthening the Reporting of Observational Studies in Epidemiology (STROBE) statement: guidelines for reporting observational studies. *J. Clin. Epidemiol.* **61**, 344–349 (2008).
